# A Comprehensive Review of *C. capsularis* and *C. olitorius*: A Source of Nutrition, Essential Phytoconstituents and Pharmacological Activities

**DOI:** 10.3390/antiox11071358

**Published:** 2022-07-12

**Authors:** Ashok Biswas, Susmita Dey, Siqi Huang, Yong Deng, Ziggiju Mesenbet Birhanie, Jiangjiang Zhang, Delara Akhter, Liangliang Liu, Defang Li

**Affiliations:** 1 Annual Bast Fiber Breeding Laboratory, Institute of Bast Fiber Crops, Chinese Academy of Agricultural Sciences, Changsha 410205, China; susmita.ag4sau@gmail.com (S.D.); huangsiqi@caas.cn (S.H.); dengyong@caas.cn (Y.D.); zegje23@gmail.com (Z.M.B.); zhangjiangjiang6@163.com (J.Z.); 2 Department of Agronomy, Zhejiang University, Hangzhou 310027, China; delara@zju.edu.cn; 3 Department of Genetics and Plant Breeding, Sylhet Agricultural University, Sylhet 3100, Bangladesh; 4 Department of Chemistry, Institute of Bast Fiber Crops, Chinese Academy of Agricultural Sciences, Changsha 410205, China; liuliangliang@caas.cn

**Keywords:** *Corchorous capsularis*, *Corchorous olitorius*, bioactive compound, traditional uses, biological activities

## Abstract

Plant bioactive compounds have gained global significance in terms of both medicinal and economic ramifications due to being easily accessible and are believed to be effective with fewer side effects. Growing relevant clinical and scientific evidence has become an important criterion for accepting traditional health claims of medicinal plants and also supports the traditional uses of *Corchorus* as folk medicine. *C. capsularis* and *C. olitorius* have broad applications ranging from textile to biocomposite, and young leaves and shoots are used as healthy vegetables and have long been used as traditional remedies for fever, ascites, algesia, liver disorders, piles, and tumors in many cultures. This review systematically summarized and emphasized the nutritional attributes, mostly available bioactive compounds, and biological and potential pharmaceutical properties of *C. capsularis* and *C. olitorius*, disclosed to users and non-users. Results suggest that various phytochemicals such as cardiac glycosides, phenols, flavonoids, sterols, lipids, and fatty acids were found or analytically identified in different plant parts (leaf, stem, seed, and root), and many of them are responsible for pharmacological properties and their antitumor, anticancer, antioxidant, antinociceptive, anti-inflammatory, analgesic, antipyretic, antiviral, antibacterial, anticonvulsant, antidiabetic and antiobesity, and cardiovascular properties help to prevent and cure many chronic diseases. In addition to their use in traditional food and medicine, their leaves have also been developed for skin care products, and some other possible uses are described. From this review, it is clear that the isolated compounds of both species have great potential to prevent and treat various diseases and be used as functional foods. In conclusion, this comprehensive review establishes a significant reference base for future research into various medical and functional food applications.

## 1. Introduction

Plants have provided nourishment and other health benefits to humans and animals for as long as they have existed. According to reports, around 80% of the world’s population relies on natural products and plant-based medicines as their primary source of medication [1], and about 25% of all medications are derived from 500 various herbal plant species [2]. People are becoming more aware of the side effects of synthetic drugs, as well as high cost, unavailability, inaccessibility, and unexpected loss of efficacy, so there is a growing interest in these traditional medicines [3]. The rising costs of health care and mainstream medicine’s failure to treat certain diseases have also resulted in the increased use of traditional medicine in disease prevention. So, the scientific community is searching to find an alternative source such as a plant to develop a more stable and efficient drug to treat various diseases.

**Figure 1:**
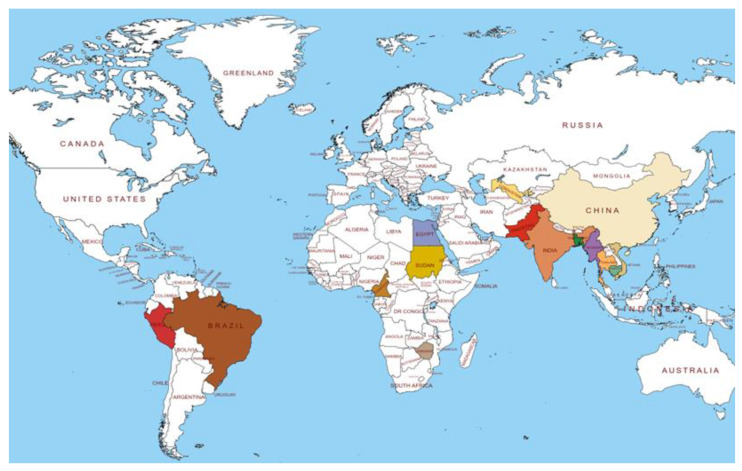
Jute-producing countries in the world.

*Corchorous* is a genus of annual herbs belonging to the Tiliaceae family that contains about 50–60 species, but only two species, *C. olitorius* and *C. capsularis*, are well-known for commercially bast fiber production and are distributed throughout the world’s tropics, subtropics, and warm-temperate regions [4,5]. The major jute-growing countries are India, Bangladesh, China, Thailand, and Nepal (Figure 1). In jute-producing countries, short and branched stems with mucilaginous leaves of these species are used as vegetables in foods to promote community [6,7]. In addition, the traditional uses of *corchorus* as folk and ayurvedic medicine to treat a variety of human diseases has been made evident, as more people recognize natural products are also effective, safe, non-narcotic, and affordable, without any kind of side effects [8].

**Figure 2:**
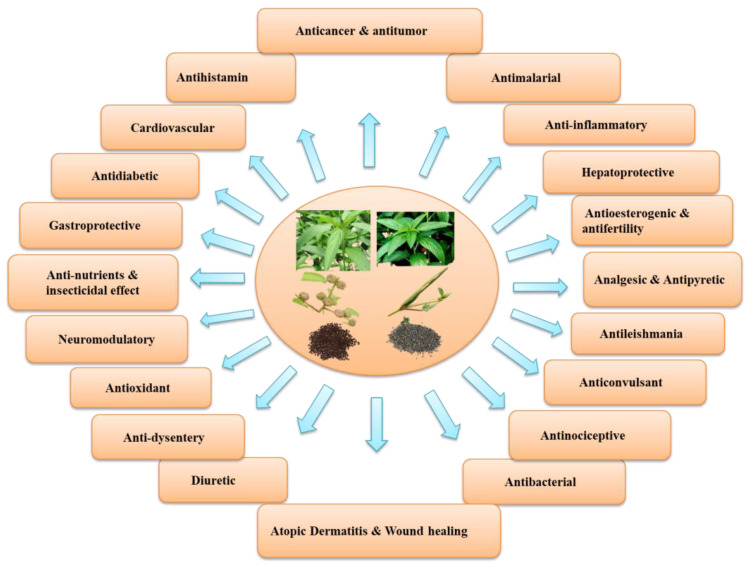
An overview of various biological, pharmacological, and medicinal attributes of *C. capsularis* and *C. olitorius*.

It has been reported that the leaves of edible species of *Corchorus* are an excellent source of protein, vitamins, and certain hormone precursors and are rich in minerals [9,10]. In addition to the micro and macronutrients, it contains a wide range of bioactive compounds such as glycosides, phenolics, flavonoids, tannins, saponins, sterols, triterpenoids, ionones, fatty acids, and carbohydrates, all of which have been reported from different the plant parts and seeds of two commonly cultivated species. These substances have powerful antipyretic, diuretic, analgesic, antioxidant, antimicrobial, and antitumor properties [11]. In addition, the leaves of *C. capsularis* and *C. olitorius* were reported to exhibit protective effects, mainly antitumor and anticancer promoting activity as well as antioxidant, anti-inflammatory, antitumor, antifungal, gastroprotective, antinociceptive, analgesic, and antibacterial activities [12,13,14] (Figure 2). Moreover, both species inhibit α-glucosidase and α-amylase activities [15] and are linked to a reduction in oxidative stress and an increase in *β*-oxidation in the liver [16].

Despite therapeutic and prophylactic uses, little is known about *Corchorus* leaf-derived active principles, their biological roles, and toxicological limits. Several previously published reports have provided fragmentary summaries of morphological, phytochemical, and ethnomedicinal properties of *C. capsularis* and *C. olitorius* in general. Such information is crucial for achieving the potential commercialization of leaves as well as different parts of both species. However, a systematic review of the literature that is organized, comprehensive, and targeted has not been conducted to assess the complete attributes of the nutritional, bioactive compounds and pharmaceutical properties of *C. capsularis* and *C. olitorius*. Therefore, this article seeks to fill this gap by presenting a comprehensive and up-to-date overview of the nutritional and phytochemical profiles, and biological as well as pharmacological features, of both species. For this, an unbiased literature search was conducted using several well-known scientific search engines with comprehensive coverage for a literature search, including Google, Google Scholar, ResearchGate, PubMed, Web of Science, and Scopus using different combinations of the following: *C. capsularis*, *C. olitorius*, nutrient, phytochemistry, ethnomedicine, health effects, biological activities, pharmacological activities, in vitro, and in vivo. The authors did not attempt to contact the investigators or identify any unpublished data. A detailed bibliographic search was carried out, and information was examined, critically analyzed, and collated systematically to ensure the reproducibility of the literature review process. This review aims to take advantage of the key findings concerning phytoconstituents, and emphasizes their therapeutic potential based on pharmacognosy, traditional medicine, and experimental knowledge. Another aim is to identify the information gaps and provide a valuable guide for future scientific research and the pharmaceutical industry as well as contributions to the field.

## 2. Use in Local and Traditional Food and Medicine

*Corchorus* spp., especially the leaves of *C. olitorius* and *C. capsularis*, are used as leafy vegetables in many Asian, African, and European countries [4,17,18,19] (Table 1). It is simple to prepare and may be incorporated in a variety of dishes to enable people to take advantage of its vitamins and minerals. Due to their mucilaginous texture, the leaves have a viscous consistency and are widely consumed as soup in Middle Eastern countries. The leaves are used as a condiment in Bengal, where it is considered a tonic, and frequently incorporated into rice for the daily diet. Dried leaves are used in herbal tea, while seeds are used as a flavoring agents [20].

The dried leaves are a favorite of the Boros of northeast India, who make narji, a mucilaginous preparation made with fatty pork and lye. Most often, it is lightly sauteed and eaten with rice or rice gruel in Sambalpur and the western part of Odisha in summer. The leaves of *C. olitorius* are commonly consumed together with bamboo shoots in the Philippines, whereas in Thailand they are eaten with plain rice congee and tastes like spinach and samphire [8,21]. In Vietnamese cuisine, it is known as rau đay and is made into a soup with shrimp. The Luhya people of Western Kenya eat jute leaves with starchy foods such as ugali, a staple food for most Kenyan communities. Nigerian cuisine is usually used as a condiment for other starchy foods such as amala, cassava, yam or millet as well as used in ethno-medicine to facilitate labor and smooth child delivery, especially in the Yoruba people [22]. In addition, some tribes of Nigeria used leaf extract to treat a menstrual disorder that is associated with excessive uterine contractions during menstruation [23] and to arrest threatened miscarriage and induce tocolysis [24]. Moreover, its leaf decoctions are used to treat iron deficiency and folic acid deficiency and in the treatment of anemia. In many West African cooking traditions, the leaves are used as an ingredient to make a traditional slimy soup or sauce. It has been recognized and popular as an Egyptian national dish in Egypt. In Turkey and Cyprus, it is typically cooked into a kind of chicken stew and flavored with lemon and olive oil [25]. Mallow leaf stews with rice are a known cuisine of the Middle East [8,26]. Recently, molokhia leaves were also used to develop sushi wraps as a promising viable substitute for Nori [27,28]. The young leaves of *C. capsularis* are the most edible part, mainly consumed in a salad or cooked as a pot herb, as they have been in many parts of the world for a long time. In Japan, leaves of both species were regarded as healthy food, and young dried leaves are considered an alternative to coffee or tea and a thickener in soups [29,30].

Consumption of the *C. olitorius* and *C. capsularis* leaves are reported to be a diuretic, demulcent, deobstruent, purgative, refrigerant, carminative, bitter tonic, lactagogue, and blood purifier; the leaf twigs are used for cardiac problems and the stems for treating cardiovascular disorder [31]. The seeds are used as a laxative and the roots for treating toothache, and a decoction of the roots is used as a tonic to increase strength. In Tanzania, leaf infusions are used as a tonic, appetite stimulant, and to treat constipation [32,33]. Both of these plants’ leaves are used in ethnomedicine to treat aches and pains, swellings, cystitis, dysuria, gastroenteritis, dysentery, malaria, enteritis, fever, gonorrhea, diabetes, dyspepsia, liver disorders, pectoral pains, and tumors [29,30,33,34,35,36]. *C. olitorius* is one of the plants used in folk medicine to treat measles [37]. Furthermore, it is also a component in facial moisturizers, lotions, hair tonics, and hand creams [38].

## 3. Nutritional Composition of Jute

Edible species of *Corchorus*, especially *C. olitorius* and *C. capsularis*, are used as a vegetable in jute growing as excellent sources of proteins, dietary fiber and carotene, and vitamins (A, C, E), and they are also rich in mineral nutrients such as K, Ca, P, and Fe [9,10] (Table 2). In addition to this, *C. olitorius* content has a high amount of iron, folate, amino acids, and essential minerals [39], which are essential for preventing anemia [9]. It has been reported that *C. olitorius* leaves contain a significant amount of 17 macro and micronutrients, including carbohydrates, protein, fat, fiber, calcium, phosphorous, potassium, ascorbic acid, beta-carotene, riboflavin, thiamine, niacin, etc [8]. Regarding the micronutrients, the leaves of both species had a high concentration of Fe and Se compared with other leafy vegetables, whereas Zn and Se content varied from 0.91–7.35 mg/100 DW and 85.51–168.85 μg/100 g DW, respectively [40]. This variation may be the genetic variations among the cultivars and environmental growing conditions. A number of studies reported that *C. olitorious* and *C. capsularis* were found to be crude protein (14%, 3.86%), potassium (44.73 mg, 40.43 mg), and beta-carotene (8.13 mg, 6.13 mg) per 100 g, respectively [8,41]. It was stated that beta-carotene is a group of phytochemicals that are liable for different colors of the fruits and vegetables, playing a vital role in the prevention of mortal disease and that *C. olitorius*’ content is higher than other *Corchorus* spp. as well as other leafy vegetables [42]. It was found that 100 g of *C. capsularis* leaves contained 80.4–84.1 g H_2_O, 43–58 calories, 4.5–5.6 g protein, 7.6–12.4 g carbohydrate, 0.3 g fat, 1.7–2.0 g fiber, 2.4 g ash, 266–366 mg Ca, 97–122 mg P, 7.2–7.7 mg Fe, 12 mg Na, 444 mg K, 6.41–7.85 mg beta-carotene equivalent, 53–80 mg ascorbic acid, 0.26–0.53 mg riboflavin, 0.13–0.15 mg thiamine, and 1.1–1.2 mg niacin and folic acid content was considerably higher compared with other folacin-rich vegetables [43]. It was reported that jute leaves as saluyot (boiled/100 g) contain 43–58 of food energy (cal), 4.5–5.6 g of protein, 1.7–2.0 g of fiber, 2.4 g of ash, 7.6–12.4 g of total carbohydrates, 97–122 mg of phosphorous, 11.6 mg of iron, 12 mg of sodium, 444 mg of potassium, 6390 of Vit-A (I.U), 15 mg of thiamine, 28 mg of riboflabin, 1.5 mg of niacin, and 95 mg of ascorbic acid [8]. In addition, it was reported that both the raw and cooked fruit of *C. olitorius* composed appreciable amounts of protein, fat, fibre, ash, carbohydrate, and energy [44]. It stated that the boiled leaf saluyot of *C. olitorius* contains higher ascorbate than fresh leaves. A group of researchers identifies the leaves contained ascorbic acid (258 mg/100 g) and α-tocopherol (14 mg/100 g) [45]. The findings suggest that ascorbic acid presence in leaves might greatly contribute to the antioxidant activity and, regularly consumed, may provide enough to meet the recommended daily allowance of 60 mg for adults [46].

## 4. Bioactive Compounds in Jute

### 4.1. Polyhenols

**Figure 3:**
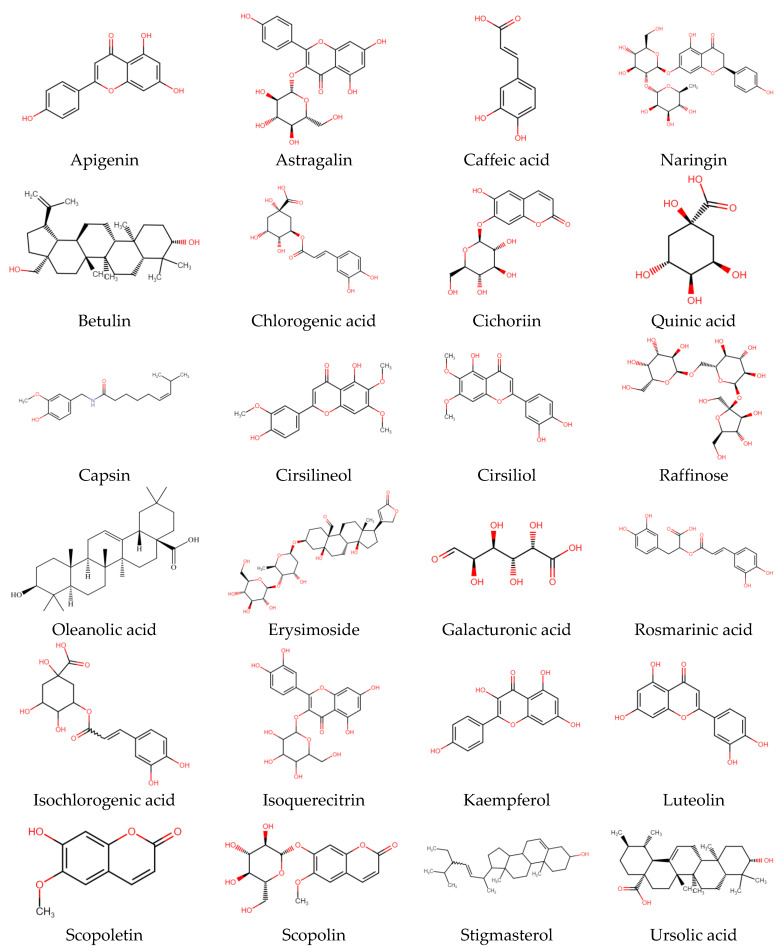

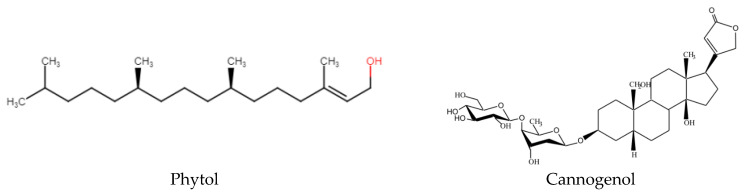
Chemical structures of the most common bioactive compound available in *C. capsularis* and *C. olitorius*.

Phenolic compounds are a large class of plant secondary metabolites that are important for the quality of plant-based foods, and along with other dietary reducing agents, are regarded as antioxidants. They protect the tissues of the body from oxidative stress-related pathologies such as as cancers, inflammation, and coronary heart disease. Phenolic acids are cyanidin and cyanidin glucosides from the bark and leaf of *C. capsularis* whereas astragalin, tolifolin, isoquercitin, and jugulanin come from *C. olitorius* leaves, as reported by researchers [47,48]. From the leaves of *C. olitorius*, a number phenolic compounds such as chlorogenic acid (383.9 mg/100 g FW), 3,5-dicaffeoylquinic acid (102.1 mg/100 g), quinic acid, gallic acid, protocatechuic acid, 4-*O*-caffeoylquinic acid, caffeic acid, 1,3-di-*O*-caffeoylquinic acid, feruloyl-quinic acids, and 4,5-di-*O*-caffeoylquinic acid were identified [47,49]. According to the literature, chlorogenic acid may be the most predominant antioxidant [45,47,50], whereas caffeoylquinic acids and dicaffeoylquinic acids have been reported to inhibit human low-density lipoprotein oxidation in vitro; there are limited studies investigating the effects in vivo [51,52]. Total phenols (9.20 mg GAE/g DW) were reported in *C. olitorius* from ethanolic acid, and recently new phenolic compounds such as trans-ferulic acid, *p*-coumaric acid, and rosmarinic acid were detected from the *C. olitorius* [41,53]. In a separate study, it was recorded that the most abundant polyphenolic compound was caffeic acid, trans-ferulic acid, rutin hydrate, ellagic acid, and quercetin hydrate in the leaves of *C. capsularis* and *C. olitorius* [54]. Most common bioactive compounds available in *C. capsularis* and *C. olitorius* and their chemical structure are illustrated in Figure 3, and isolated compounds from different plant parts with extraction technology are presented in Table 3.

### 4.2. Flavanoids

Flavonoids are biologically important compounds with high antioxidant properties that protect cells from free-radical damage caused by reactive oxygen species (ROS) and have antioxidant effects connected to several diseases, including cancer, atherosclerosis, and Alzheimer’s disease (AD) [55,56]. The leaves of *C. olitorius* contain flavanones (naringenin, naringin), flavones (cirsilineol and cirsiliol), flavones glycosides (apigenin, apegenin-7-*O*-glucoside), tolifolin (kaempferol-3-*O*-*β*-D-galactopyranoside), astragalin (kaempferol-3-*O*-*β*-D-glucopyranoside), and jugulanin (kaempferol- 3-*O*-β-L-arabinopyranoside) [47,53]. A number of researchers have reported that flavonoid glycosides, flavonol glucosides astragalin, quercetrin, isoquercitin, quercetin 3-galactoside, quercetin 3 glucoside, quercetin 3-(6 malonylglucoside) and quercetin 3-(6 malonylgalactoside), quercetin 3-*O*-galactoside (hyperoside), and catechin were identified from the leaves of *C. olitorius* [49,50,53,56,57]. The content of quercetin glycosides in *C. olitorius* leaves was 233 mg/100 g of FW, almost twice that of onion, another significant source of quercetin glycosides [52]. Authors have isolated scopoletin and fusidic acid from *C. capsularis* [58], and ethanolic extract of *C. olitorius* leaves produced the highest flavonoids content (4.10 mg QE/g) [53], which was lower than mucilaginous polysaccharides (PSc) extracts (6 mg QE/g) conducted by Yakoub et al. (2020) [41].

### 4.3. Cardiac Glycosides and Their Aglycones

Cardiac glycosides are a class of organic compounds that enhance the output force and rate of contraction of the heart by inhibiting the Na^+^/K^+^ ATPases in cardiac myocytes and are employed in heart failure and arrhythmias treatment. Several glycosidic compounds such as capsularin, chorchoritin, and corchularin have been isolated from different *Corchorus* spp. as well as a variety of algycones, including corchsugenin, corchortoxin, and corchorgenin [48]. The chemical identification of these aglycones as strophanthidin 1, the well-known aglycone of the cardiac glycoside strophanthin, was a significant advancement. From *C. capsularis* and *C. olitorius*, two digitalis glycosides have been derived, such as corchoroside A2 (mp 188–190⁰, [α]_D_^20^ = +11° (MeOH)) and corchoroside B15^20^ (mp 222–24°, [α]_D_^20^ = +68°), respectively [48]. Researchers also identified and confirmed the presence of a monoglucoside of corchoroside A in *C. olitorius* seeds, as well as a diglucoside and a triglucoside of corchoroside A [29,48]. After enzymatic hydrolysis, the seed extract of *C. capsularis* and *C. olitorius* gave a fair yield of corchoroside A. In addition to erysimoside in the seeds of *C. olitorius*, there is the presence of cardenolide glycoside, trioside of strophantidin, which has a similar structure to the polar glycoside of *C. capsularis*. A rare cardiac glycoside such as corchoroside-A and cannogenol is found in the roots extract of *C. capsularis* [58]. Among the numerous components of Vietnamese *C. olitorius* leaves, researchers first detected and identified four ionone glucosides called corchoionosides A, B, and C, (6*S*,9*R*)-reseoside, a monoterpene glucoside betulalbuside A, two flavonol glucosides isoquercitin and astragalin, and two coumarin glucosides, cichoriine and scopolin [36], whereas new coumarin 4,7-dihydroxycoumarin had been isolated from the chloroform extract of defatted seeds of *C. olitorius* [47,59]. *C. capsularis* leaves resulted in glycosides, capsulason, corchorol, capsularol, and a few sugar fragments, namely glucose, galactose, and arabinose-free sugars when treated with 4% KCl. Acid hydrolysis of capsularol produces glucose and an aglycone, capsularogenin [48]. A polar glycoside was obtained from the seeds of *C. capsularis*, and energetic hydrolysis resulted in glucose, while mild acid hydrolysis yielded aglycon strophanthidine, whereas controlled enzymic hydrolysis with β-glucosidase formulated olitoribose, glucose, and boivinose, implying that the sugar residue is gluco-olitoribose [48].

It was stated that cardenolide glycosides such as olitoriside, erysimoside, corchoroside A, and coroloside were isolated from the seeds of *C. olitorius*; helveticoside was also isolated from autofermented seeds, and deglucocoroloside was identified as an artifact through hydrolysis of the extract of seed [60]. Three new cardenolide glycosides were discovered by a group of researchers, and their structures were established based on chemical and spectroscopic evidence as cannogenol 3-*O*-*β*-D glucopyranosyl-(1→4)-*O*-*β*-D-boivinopyranoside, periplogenin 3-*O*-*β*-D-glucopyranosyl-(1→4)-*O*-*β*-D digitoxopyranoside, and digitoxigenin 3-*O*-*β*-D-glucopyranosyl-(1→6)-*O*-*β*-D-glucopyranosyl-(1→4)-*O*-*β*-D-digitoxopyranoside, as well as some new cardiotonic oligoglycosides [60]. It has been stated that dark grayish-green seeds contain a greater concentration of cardiac glucoside than dark greyish-green or yellowish-green seeds [60]. Digitoxigenin glycosides, coroloside, and glucoevatromonoside, as well as strophanthidin glycosides (erysimoside, olitoriside, biosides, trioside, corchoroside A, and helveticoside), were isolated from the methanolic extract of seeds, which were the major cardiac glycosides [60,61].

Polar glycosides A and B were produced by chloroform–butanol (1:3) fractions of *C. capsularis* seeds, while glycoside B and a new polar glycoside C were produced by chloroform–alcohol (2:1) extract. One study on *C. olitorius* seeds isolated and identified strophanthidin, strophanthidol, corchoroside A, helveticoside, and olitorin [48].

### 4.4. Triterpenoids

Triterpenoids are compounds with a carbon skeleton based on six isoprene and promising agents for the prevention of diabetic complications as well as hepatoprotective, cardiotonic, sedative, and tonic effects. Corosic acid (C_30_H_44_O_6_) was yielded when triterpenoid corosin isolated from *C. capsularis* root was refluxed with HCl [62]. A group of researchers, isolated urosolic acid, corosolic acid, betulinic acid, and oxo-corosin in fresh, undried roots of *C. capsularis* and *C. olitorius* [58,62]. Capsugenin (30-*O*-glucopyranoside), one more triterpine glucoside, was isolated from the mature leaves [63,64]. It also was reported that Egyptian origin *C. olitorius* leaves have been found to contain oleanolic acid [65]. A new dammarane triterpine glycoside, a capsin bitter-tasting substance of the leaves was identified as the 3-glucoside of 20,24-epoxy-3*β*,12*β*,25, 30-tetrahydroxydammarane (capsugenin). Later, another new triterpene glucoside, capsugenin 30-*O*-glucopyranoside, was confined from the leaves of *C. capsularis* [64,66,67]. The roots of both *C. capsularis* and *C. olitorius* were reported to contain three ursane triterpenes: corrosion, corosolic acid, and ursolic acid.

Both *C. capsularis* and *C. olitorius* roots have been reported to possess three ursane triterpenes: corrosion, corosolic acid, and ursolic acid [66]. Ramadevi (2013) stated that terpenoids such as betulinic acid and oleanolic acid were found in the roots of *C. capsularis* [58].

### 4.5. Organic Acid/Essential Oils

Chemical compositions of the essential oils of *C. olitorius* and *C. capsularis* leaves resulted in the identification of 11 and 21 compounds, with total concentrations of 24.7 and 62.9%, respectively, while 29 and 19 compounds were still unidentified for both species. It was also observed that edrane-5-one (17.7%), followed by methyl tiglate (2.73%) and trans-phytol (0.99%) of *C. olitorius* and γ-terpinene (12.1%), followed by carvacrol methyl ether (10.01%) of *C. capsularis*, represent each plant’s major compounds, respectively [65]. In other research, it was stated that 43 components were identified from *C. olitorius* leaves and flowers, the most abundant of which were benzaldehyde (56%), methyl 4-methoxysalicylate (6.55%), and carvacrol (4.75%) [68].

### 4.6. Fatty Acid

Fatty acids are ubiquitous to all living organisms, and plants synthesize a huge variety. Due to emulsification properties, it can serve as a thickening agent in cosmetic industries as well as food. It has been confirmed that the leaves of *C. olitorius* yield four higher fatty acids with a trienone function, corchorifatty acid A, B, C, and D, an undecanoic acid, a trihydroxy fatty acid, and corchorifatty acid E and F [36,69]. It was reported that there is the presence of glyceryl monopalmitate in the leaves of Egyptian *C. olitorius* [65], where β-sitosteryl fatty acid is isolated from the stem [45]. Yousef investigated the dry oil of the leaves and stem of *C. olitorius* by GC-MS and claimed both were high in hydrocarbons and fatty acids, but the leaves contained a higher concentration of hexadecanoic acid (28.52%) and 2,4-di *tert*-butyl phenol (15.01%) as a main component, whereas stem dry oil revealed ethyl palmitate (26.19%), 2,4-di *tert*-butyl phenol (14.35%), 1-eicosanol (8.27%), tetratetracontane (7.02%), 8-heptadecene (5.33%), and hexatriacontane (5.16%) in high quantities [70].

The author stated that fatty acid methyl esters of the leaves of *C. olitorius* are rich in ω3-octadecatriene fatty acid or α-linolenic acid (49%) [71], where seeds contained linoleic acid, linolenic acid, oleic acid, palmitic acid, stearic acid, and behenic acid. Linoleic acid, one of the two essential fatty acids for human health, is required for the immune system to function properly and help regulate blood pressure. A group of research and epidemiological studies have established the essentiality of ω-3 fatty acids for the normal development of premature infants’ retina and brain, as well as hypotriglyceridemic, anti-inflammatory, and antithrombotic properties [72,73]. The total fatty acid content (57.26–125.1 mg/100 g wet weight) in the leaf development stages of *C. olitorius* were found to be the highest 50 days after being sown [31]. In newer research, different parts of C. *olitorius* contained six fatty acids, namely palmitic acid, linoleic acid, heptadecanoic acid, oleic acid, steric acid, and arachidic acid. Among these, stearic acid represented the major compound in the case of the seeds (49.48%), followed by the roots (68.80%) and the leaves (58.67%), whereas palmitic acid (59.94%) was the major compound in the stems [65]. Previous research has demonstrated that seed fats of both species contain a number of fatty acids, where linoleic acid was more prominent, and *C. capsularis* seed fat is therefore, to some extent, similar to cotton seed oil, whereas in some respects, *C. olitorius* is more like sunflower-seed oil [74].

In another research, it was reported that the total fatty acids of *C. capsularis* were found to be 0.02 and 0.03% (DW) in the seeds and the vegetative parts, respectively, whereas palmitic acid was represented the most abundantly in the seeds (81.68%) and the vegetative part (54.10%) [75]. In addition, both the seeds and vegetative parts contained 87.70 and 79.62% saturated fatty acids, and 12.30 and 22.38% unsaturated fatty acids.

### 4.7. Lipid Composition

Lipids are the basic unit of membranes and our bodies’ energy source, as small hydrophobic molecules are excellent chemical messengers between organelles and cells. A group of researchers found a total lipid content ranging from (16.75–23.10 g/100 g) and lipid-like TAG (Triacylglycerol), DAG (Diacylglycerol), FFA (Free fatty acids), and PTS (Phytosterol) was determined from Thai jute cultivars’ seed oil using Iatroscan (TLC/FID), where four types of lipids significantly differed. This variation is caused by inherent differences between varieties and/or the environment in which the seed grew. A wide range of DAG concentrations (9.49%) was observed in jute seed oil, where the highest DAG was similar to that in cottonseed oil [72]. It was reported that lipids in diacylglycerol (DAG) form were found to significantly lower serum triacylglycerol levels [76,77,78]. To explain the mechanism, researchers propose that dietary DAG hypotriacylglycerolemia is caused by decreased intestinal re-esterification and chylomicron assembly or decreased chylomicron circulation secretion [78].

Triacylglycerol was the most abundant lipid in jute seed oil, accounting for 70% to 74% of the total, with phytosterol (12% to 28%) and diacylglycerol (0% to 9%) as minor components. Linoleic acid (18:2n-6), which accounts for 40–67% of total fatty acid, was the most abundant polyunsaturated fatty acid (PUFA), α-linolenic acid (18:3n-3). The findings indicated that jute seed oil could be a source of edible PUFAs [72].

### 4.8. Phytosterols and Hydrocarbons

Sterols are a subclass of steroids with the formula C_17_H_28_O that contain a hydroxyl group at the 3-position of A-ring. Hasan and Kadhim (2018) found total sterol content was 1.8, 1.6, 1.4, and 0.6% (DW) in the seeds, leaves, stems, and root petroleum ether extract of *C. olitorius* and 0.05 and 0.12 (DW) in the seeds and vegetative parts of *C. capsularis*, respectively [75], which was higher than earlier research [79]. In a recent study, β-sitosterol, a novel antileishmanial agent, was isolated and fully characterized based on spectroscopic analysis (FTIR, H NMR, C NMR, and GC–MS) from the chloroform extract of *C. capsularis* [80]. It has been reported that steroids such as β-sitosterol and β-sitosterol-D-glucoside have been isolated from the leaves and roots of both species, with β-sitosterol 3-*O*-*β*-D-glucoside from the Egyptian origin *C. olitorius* leaves [65] and the roots of *C. capsularis* [48,81].

Estimation of hydrocarbon contents showed that octadecane is the major compound in the roots (55.54%), followed by the stems (13.81%) and leaves (5.12%), and a large quantity of tetradecane was recorded in the seeds (33.58%), followed by the roots (22.79%). The investigated parts of *C. capsularis* found that the seeds are composed mainly of cholesterol (28.52%), whereas the vegetative part is composed of cholesterol (12.46%), campasterol (24.98%), stigmasterol (4.38%), and β-sitosterol (10.39%). Hydrocarbon contents of the examined parts of *C. capsularis* showed that octadecane is the major compound in the seeds (45.13%) and heptadecane was only present in the vegetative part [65].

Several researchers investigated unsaponifiable fractions by GLC and showed that the seeds and roots contain mainly cholesterol and the triterpenoid compound β-amyrin, where cholesterol, β-sitosterol, and β-amyrin were detected from the leaves. It was noticed that cholesterol represents the major compound in the leaves (19.28%), whereas β-amyrin represents the major compound in (23.18%) the stems. β-Sitosterol is present in stems (8.39%) and leaves (7.62%), in contrast, stigmasterol is only in the stems (9.37%) [65].

### 4.9. Polysaccharides and Other Sugars

Polysaccharide has been reported from the mucilage extract of dried leaf of C. olitorious; it is rich in uronic acid (65%) and contains glucose, rhamnose, galacturonic acid, and gluconic acid in a molar ratio of 1.0:0.2:0.2:0.9:1.7, as well as the acetyle group [20]. In the leaves of *C. olitorius*, monosaccharide analysis revealed that galactose was predominant, followed by rhamnose, glucose, arabinose, and mannose, with the least amount of glucuronic acid [41]. Compared with the latest results, galactose, arabinose, and mannose were not detected in earlier research. The seed of *C. capsularis* and *C. olitorius* comprised free sugars, glucose, sucrose, fructose, raffinose, arabinose, and galactose, while the root extract consisted of glucose and fructose arabinose and raffinose [62], while fructose and galactose were also identified in the leaves and bark. This polysaccharide promotes the proliferation of murine splenocytes. The leaves of *C. capsularis* and *C. olitorius* contain two active components: monogalactosyldiacylglycerol (1,2-di-*O*-*α*-linolenoyl-3-*O*-*β*-D-galactopyranosyl-*sn*-glycerol) and phytol (3,7,11,15-tetramethyl-2-hexadecen-1-ol) [82], and concentration increased with hot water treatment.

### 4.10. Volatile Compounds

It was stated that a total of 45 and 49 components were identified in natural fresh leaves and cupric chloride-treated leaves of *C. olitorius* by GC-MS analysis, respectively. In fresh leaves, *cis*-3-Hexen-1-ol, *cis*-4-hexen-1-ol, terpinolene, sabinene, and phytol were the major compounds, whereas the major compounds found in treated leaves were *cis*-4-hexen-1-ol, *cis*-3-hexen-1-ol, tetradecanal, and phytol [83]. In a another study, five compounds were identified as scopoletin, isopimpinellin, xanthotoxol, fraxinol, and peucedanol and showed a strong antimicrobial activity [84].

## 5. Biological Activity

### 5.1. Antitumor and Anticancer Promoting Activity

Recent epidemiological studies have found that consuming a lot of plant leaves and fruits may be linked to a lower risk of cancer, particularly in the gastrointestinal tract. Two active components named phytol (a side chain of chlorophylls) and mono-galactosyldiacylglycerol have been identified in the leaves of *C. capsularis* and *C. olitorius* [82]. Reportedly, phytol and mono-galactosyldiacylglycerol at concentrations 15 g/mL and 30 g/mL completely inhibited the induction of EBV early antigen, respectively. Earlier, it was stated that phytol’s cytotoxicity on cancer cells was caused by apoptosis induction, whereas mono-galactosyldiacylglycerol inhibits the mammalian DNA polymerases activities, including DNA polymerase β related to repair, and made a strong apoptosis in gastric cancer cells. In a study by Li et al. (2012), human hepatocellular carcinoma (HepG2) cells treated with *C. olitorius* ethanol extract (ECO) at 12.5 µg/mL concentration demonstrated a significant reduction in cell viability, and by increasing caspase-9 activity and cytochrome c leakage from mitochondria with reduced membrane potential indicated triggering apoptosis, while normal FL83B hepatocytes were unaffected. Thence, apoptosis in ECO-treated HepG2 cells through caspase-dependent mitochondrial pathway may be induced partially by phytol and mono-galactosyldiacylglycerol [14]. In the evaluation of nutraceutical properties, polyphenol-enriched extracts (PEEs) of *C. olitorius* showed cytotoxicity against the colorectal Caco-2 cell line by triggering oxidative stress without disruption of the healthy CCD841 line and inhibition of the activity of glutathione-independent antioxidant enzymes [49]. Therefore, *C. olitorius* is suggested as a foodstuff plant with the potential to develop chemopreventive agents for human cancers.

In research, the cytotoxic inhibitory potential of *C. olitorius* seed extract and the genotoxic potential of the leaf, as well seed extract, were displayed on human multiple myeloma-derived ARH-77 cells in a dose-dependent manner [91]. This finding is supported by Newman et al. (2003), who reported that medicinal plants can exert a significant anticarcinogenic effect [92]. Similarly, the potential protective effects was observed against the foodborne mycotoxins of AFB_1_ and/or FB_1_ in H4IIE-luc rat hepatoma cells, where *C. olitorius* aqueous extract treated cells were statistically more viable cells and had less DNA damage in H4IIE-Luc cells exposed to mycotoxins than not treated with plant extract. This might be due to the abundant presence of flavonoid compounds and the antioxidant capacity [93]. Therefore, the result suggests that the aqueous extract of *C. olitorius* possesses cancer chemo-preventative agents.

In a newer research, it was recorded that chlorogenic acid (CGA) and isoquercetin (IQ) derived from the aqueous extract of *C. olitorius* significantly inhibited proliferation in vitro of human melanoma (A-375), gastric cancer (AGS), and pancreatic cancer (SUIT-2) cells and suppressed the growth of A-375 and AGS tumor xenografts in a CAM model by inducing apoptosis and antiangiogenetic properties [94]. Soykut et al. (2018), found that aqueous extract of *C. olitorius* leaf effectively inhibits both primary (Colo-320) and metastatic (Colo-741) colon adenocarcinoma cells at 50 µg/mL concentration in the case of in vitro anticancer and apoptotic induction by increasing caspase-3, cytochromec, and FasL immunoreactivity significantly in Colo-741 cells, indicating activation of extrinsic and intrinsic apoptotic pathways and being more effective in metastatic colon adenocarcinoma cell lines [12]. These apoptopic induction properties are due to the presence of natural anticancers such as quercetin, isoquercetin, and chlorogenic acid in the extract. Quercetin induction of apoptosis by increasing intracellular ROS, as well as increasing Bax protein expression and inhibiting Bcl-2 in HCT-116 via the intrinsic pathway, has been reported. Chlorogenic acid had cytotoxic effects in HT-29 colon cancer cells by increased caspase-3, while decreasing Bcl-2 activity and suppressing heat shock protein 70. As a result, tumor growth was inhibited [95]. Thus, the results indicate that extracts may have anticancer properties and may be used as a guide for treating colon cancer with phytomedicines rather than chemotherapy. Researchers have established the cytotoxicity of two identified compounds named Methyl-1,4,5-tri-*O*-caffeoyl quinate and trans-3-(4-Hydroxy-3-methoxyphenyl) acrylic anhydride (a popular chemo-preventive curcumin in cancer therapy) against HeLa, HL460 lung cancer cell line, and PC3 prostate cancer cell line [13]. When tested at concentrations up to 1.6 mM, the compounds were found to have mild cytotoxic activity against HeLa cells at ≥800 mM. A study determined the cytotoxicity of the different extracts from *C. capsularis* leaves by Brine shrimp lethality bioassay; the result reveal that butanol extract showed the most potent 71.14% inhibition at a concentration of 1.25 mg/mL, followed by ethyl acetate and methanol with inhibitions of 28.57% and 14.28%, respectively [96]. Based on the above discussion, it can be concluded that *C. olitorius* may be a source of naturally occurring “lead” compounds with antitumor activity. A summary of the biological activities of *C. capsularis* and *C. olitorius* are presented in Table 4.

### 5.2. Gastroprotective Effect

A peptic ulcer is an ulcer of one of the areas of the gastrointestinal tract that occurs as a result of excess acid, a breakdown in mucosal defenses, and peptic activity in gastric juice [97]. The results obtained by researchers point out that an extract of *C. olitorius* leaf demonstrated potential antiulcer activity in rats by decreasing acid secretion, improving mucosal defense, and increasing in vivo antioxidant status [98], where it was found that mucus productions increased dose-dependently. The highest dose (400 mg/kg) of plant extract resulted in mucus production increase up to 162.90 mg and a highly significant reduction in ulcers, with a corresponding curing rate of 94.08% by increasing the antioxidant enzymes activities. These findings indicating *C. olitorius* may accelerate ulcer healing by boosting angiogenesis, cell proliferation, migration, and maturation of the granulation tissue. A similar finding was also stated by a group of researcher, where the extract’s ability to inhibit gastric acid secretion and lesser gastric ulcer areas was found against pylorus ligation-induced gastric lesions [99,100]. Results also show that oral extract administration reduces the ulcer indices dose-dependently substantially, with significant increases in the production of gastric mucosa in rats. The decreased gastric acidity indicates that the extract’s cytoprotective mechanism, similar to that of endogenous prostaglandins, may involve either direct inhibition of gastric secretion or simple neutralization of the acid secreted by parietal cells, as well as reinforcement of the in vivo antioxidant status by the increase in SOD and a decrease in MDA. In addition to histological analysis, immunohistochemistry confirmed the protection-mediated up-regulation of Hsp70 and the down-regulation of Bax proteins. In conclusion, *C. olitorius* reduces gastric motility by reducing the area of the gastric wall of ulcerated areas and decreasing the infiltration of edema and leukocyte into submucosal layers.

### 5.3. Antinociceptive, Anti-Inflammatory, and Antipyretic

Antinociceptive activity was determined using formalin tests, writhing, and hot plate, whereas anti-inflammatory activity was found using the carrageenan-induced paw edema and antipyretic activity in brewer’s yeast-induced pyrexia tests [29,30]. Different doses of air-dried leaves extract (20, 100, and 200 mg/kg) exhibited significant (*p* < 0.05) antinociceptive, anti-inflammatory, and antipyretic activities independent of dosage. A separate study by Zakaria et al. (2005), suggested that the antinociceptive properties of *C. olitorius* are enhanced by temperature and may be mediated peripherally, rather than centrally, at least in part, via the involvement of the opioid receptor system [101]. This suggests that the heating process may enhance the breakdown of the effective compound responsible for the antinociceptive activity of COAE. It also found that the highest antinociception was achieved by 75% concentration of COAE treatment, while the highest concentration (100%) was found to produce no antinociceptive activity. This is the possibility that the loss of antinociceptive activity of the COAE for the highest concentrations of bioactive compound present in the extract causes deactivation of the antinociceptive-inducing receptors within the peritoneal cavity as a result of the presence of a high concentration of the bioactive compound of the respective extracts [101].

Similarly, Oyewole et al. (2015) provided the information that *C. olitorius* root is regarded as antipyretic and anti-inflammatory, where pyrexia and inflammation were induced by injecting 10 mL/kg of 20% (*w*/*v*) suspension of brewer’s yeast and 0.1% carrageenan, respectively [102]. The results indicate that *C. olitorius* root extract significantly (*p* < 0.05) decreased the elevated temperature following brewer’s yeast injection, and that in inflammatory tests, compared with the control group, the test group had significantly (*p* < 0.05) smaller granuloma weights and paw sizes. Thus, the above finding confirmed that *C. olitorius* root is a strong source of phytomedicine that can effectively treat inflammation and pyrexia associated with certain diseases that support its folklore use. Yan et al. (2013) reported that phenolic crude extracts *C. olitorius* significantly reduced LPS-induced protein expression of nitric oxide synthase (iNOS) and cyclooxygenase 2 (COX-2) in LPS-stimulated J774A.1, and prostaglandin E_2_ (PGE_2_) synthesis was suppressed in dose-dependent manners [103].

Recent studies have reported that *C. olitorius* leaf extracts significantly reduced the amplitudes of uterine tissue contraction dose-dependently, with the highest dose (666.67 μg/mL) achieving a 100% inhibitory effect, and significant inhibition of oxytocin-induced contractions was also seen [22]. Significant smooth relaxing effects of the uterine and gastrointestinal muscles, possibly due to their molecular interactions with receptors, inhibited contractions of the smooth muscles for extracts rich in flavonoids [104]. According to Parvin et al. 2015, a methanolic extract of *C. olitorius* aerial parts had a significant impact on reducing the number of abdominal constrictions induced by acetic acid through intraperitoneal administration [105]. It was stated that 100 mg/kg MECO was better than 200 mg/kg aspirin in relieving pain. Due to relaxing effects on the uterine, *C. olitorius* is used in ethnomedicine to facilitate labor and promote the smooth delivery of babies, particularly among the Yoruba people [106].

### 5.4. Antioxidant Activity

Free radicals are generated in large quantities due to different reactions within our bodies. In normal metabolic processes, free radicals cause pathological changes as a result of their interaction with various biological compounds both inside and outside living cells [107]. Consuming dietary antioxidants has been shown to reduce the risk of developing oxidative stress-related diseases such as diabetes, cardiovascular disease, and cancer by scavenging free radicals and reducing oxidative stress [108]. It was reported that *C. capsularis* leaves contained higher levels of total antioxidant capacity than that of *C. olitorius* leaves [54]. In the methanolic leaf extracts of both of the species, there was a higher percentage of radical scavenging activity compared with superoxide anion and DPPH radical scavenging assays, indicating that they are rich in phytochemicals with potent natural antioxidative activities [96,109]. A study by Yan et al. (2013) stated that a fraction of the whole plant, stem, and leaves showed significantly higher DPPH radical scavenging effects that are related to higher content of phenolics [103]. In addition, linoleic acid autoxidation inhibition was greater in all fractions than in α-tocopherol. A new study undertaken by Biswas et al. (2020) reported the leaves of ethanolic extract of *C. olitorius* demonstrated better DPPH, ABTS, and FRAP activities than *C. capsularis*, and a strong positive correlation exists between TFC and FRAP, followed by TPC and FRAP and TFC and ABTS [40]. Therefore, these correlations suggest that the presence of phenolic compounds such as flavonoids and polyphenols may be accountable for antioxidant activities and react with free radicals. Another study revealed that the methanolic extract of immature leaf extracts demonstrated the highest DPPH radical scavenging activities from any other growth stages that are nearly close to the value of α-tocopherol [110].

Polyphenols are frequently linked to the antioxidant capacity of the corchorous species; there are few studies on the role of other chemical components. Oboh et al. (2009) reported that polar aqueous extract exhibited significantly higher DPPH radical scavenging activity, Fe^2+^ chelating ability, and trolox equivalent antioxidant capacity than nonpolar hexane extracts [111]. The high polar polyphenols (flavonoids and nonflavonoid polyphenols) and vitamin C are responsible for the antioxidant activity of the hydrophilic extract, while the presence of carotenoids may have been responsible for the antioxidant activity of hexane extract [112]. The evaluation of the reducing power with the DPPH method revealed that the hydroethanolic extract of *C. olitorius* was the most active (IC_50_ = 45.58 µg/mL), which was in agreement with the results obtained by the FRAP method [112]. Among phytochemicals, phenolic compounds are the main contributors to antioxidant activity in plant extracts due to their high total content, interaction, redox property of an individual or combination of their diverse chemical structures, and synergistic effectiveness as hydrogen donors, reducing agents, and free radical scavengers [113,114].yos.

It was observed in the in vitro study that ethanolic extract (EE) *C. olitorius* exhibited overall better antioxidant activities (91.65% against DPPH radicals, 86.19% against β-carotene, and 70.24% against lipid peroxidation at 500 μg/mL) than the EAE and AE, dose-dependently [53]. Similar antioxidant activities were the half-maximal inhibitory concentration (IC_50_) value for DPPH, and ABTS radical scavenging activity was 37.65 µg/mL and 33.19 µg/mL, respectively, with the methanolic extract of *C. olitorius* [115]. Yakoub et al. (2020) reported that 1.5 mg/mL mucilaginous polysaccharides (PSc) extract of *C. olitorius* revealed 90%, 78%, and 69% against DPPH radical scavenging, lipid peroxidation, and β-carotene, respectively [41]. The same concentration of PSc also demonstrated an effective response against hydroxyl radicals and DNA breakage with a FRAP assay. This is consistent with previous research that reported better chelating efficiency on ferrous ions, this may due to the presence of condensed tannins at a high level [103]. Azuma (1999) reported that 5-caffeoylquinic acid showed a predominant phenolic antioxidant with stronger scavenger properties than ascorbic acid [47].

### 5.5. Cardiovascular Activity

Apart from its antioxidant capacity, the jute leaf has received a lot of attention in possible cardiovascular-protective activities. In the literature, it was reported that Corchortoxin (strophanthidin) is a cardiac, isolated from *C. capsularis* seeds, which exhibits cardiac activity comparable to digitalis genius [88]. In 2000 patients with cardiac decompensation, the clinical effects of Soviet-produced strophanthoids (cardiac glycosides with strophanthin-like effect speed, coronary-vessel effect, diastolic and diuretic effect degrees, etc.) have been studied. The most active drugs used in cardiac problems are olitoriside and corchoriside, isolated from *C. olitorius* [48]. Corchoroside A and B isolated from *C. olitorius* seeds provide digitalis-like action. The esterification of OH group in 3′ and 4′ position of Corchoroside A and in the position of 3′, 4′, and 19 of Corchorosol A by acetic acid resulted in the creation of new acetylated cardenolide. Corchorosol derivatives from *C. capsularis* are more effective than corchorosides in treating cardiovascular diseases.

In a study, the authors stated that *C. olitorius* seeds displayed strong inhibitory action against Na and K-ATPase, which correspond to digitoxin ouabain for the presence of cardenolides and oligoglycosides (corchorusosides A–E) [89]. These act on the contractile force of the cardiac muscle because of their ability to disrupt the heart function, most of them are highly toxic. Jute seed extract has shown greater activity than corchortoxin. The result of a study by Langendroff, where rabbit hearts were tested with the seed extract at different concentrations, shows that left ventricular pressure and coronary blood flow increase at low concentrations, where high doses were significantly decreased with a sharp increase in heart rates with ventricular fibrillation. Khatib et al. (1998) reported that the presences of active ingredients in the seed extract of *C. olitorius* are associated with direct effects on the cardiac myocardium. They also stated that *C. olitorius* seed oil exhibits estrogenic activity and is capable of treating cardiovascular diseases because of a high amount of active cardiac principles, particularly olitoriside, which demonstrated equivalent strophanthin effects in chronic cardiac patients [70].

According to one study by Airaodion et al. (2019), *C. olitorius* leaf extract has significant health benefits in terms of hyperglucosemia and hyperlipidaemia by significantly lowering fasting blood sugar, total cholesterol, LDL-cholesterol, and triglyceride, while significantly increasing HDL-cholesterol and HDL/LDL cholesterol ratio when compared with the control group [116]. This indicates that leaf extract is exceedingly helpful in controlling obesity and hypertension by inhibiting multiple targets and drug development from natural plant products.

It has been reported that Methyl-1,4,5-tri-*O*-caffeoyl quinate top is a ligand that is the major compound in the methanolic extract of *C. olitorius* and has a stronger bond and can block the receptor ACE2. Thus, it could make it difficult for coronavirus to enter cells, which could at least significantly slow the epidemic until the virus disappears and allow us to propose a methanolic extract of *C. olitorius*, and suggested as a natural and reliable treatment during the first stage of COVID-19 [117].

### 5.6. Antidiabetic and Bioadsorbent Properties

Plants that are rich in diverse polyphenolic compounds with the ability to interact with proteins and suppress carbohydrate-hydrolysing enzymes are important for their possible antidiabetic effect [118]. Besides antioxidant potential, there are considerable ethnomedicinal reports regarding the treatment of diabetes owning to the potential antidiabetic action of *Corchorus* spp. In a study, Oboh et al. (2012) evaluated the antidiabetic and antihypertensive inhibitory effects of soluble-free and bound polyphenol-rich *C. olitorius* leaf extracts on pancreatic α-amylase, α-glucosidase, and angiotensin I converting enzyme [119]. The result shows that extracts inhibited α-amylase, α-glucosidase (12.5–50.0 µg/mL), and ACE (10.0–50.0 µg/mL) in a dose-dependent manner and inhibitory activities disclosure by IC_50_, and free polyphenol-rich extracts had significantly (*p* < 0.05) high α-amylase, α-glucosidase, and ACE at 17.5 µg/mL, 11.4 µg/mL, and 15.7 µg/mL, respectively, in term of IC_50_. The inhibitory action of the extracts of *C. olitorius* consists of earlier studies where findings claimed that plant phytochemicals are mild inhibitors of α-amylase and powerful inhibitors of α-glucosidase [120,121], suggesting polyphenol-rich extracts of *C. olitorius* may help address a major draw-back associated with synthetic α-glucosidase inhibitors. Authors reported that all *Corchorus* species had a significant (*p* < 0.05) inhibitory effect on α-amylase and α-glucosidase activities at low concentrations (0–8 μg/mL) along with exhibiting antioxidant properties [15]. These findings can explain the biochemical justification behind the use in the management of T2DM in folklore medicine.

It has been reported that the ethanolic extract of the *C. olitorius* leaf significantly (*p* < 0.05) elevated levels of serum and liver bilirubin (direct and total), transaminases (AST and ALT), alkaline phosphatase, urea, creatinine, total cholesterol, triglyceride, and LDL-C, as well as reduced levels of total protein, globulin, albumin, and HDL-C in the alloxan-induced untreated diabetic rats normalized upon treatment with the ethanolic extract of *C. olitorius* leaf. Thus, the ethanolic extract of *C. olitorius* leaf exhibited antihyperglycemic and anti-dyslipidemic effects, and hence it could be considered safe for use as an antidiabetic recipe [122]. Another group of researchers pointed out that ethanolic seed extract of *C. olitorius* is a valuable antidiabetic agent due to its ability to lower blood glucose levels [32]. A significant difference (*p* ˂ 0.001) was observed in blood sugar levels between the control and treatment group from the 30th minute and 2nd hour in the glucose-loaded hyperglycaemic test (OGTT), where regular rats were administrated with glucose 30 min after orally administrated ethanolic extracts of *C. olitorius*. These findings show that *C. olitorius* leaves play a crucial role in health. A separate study reported that *C. olitorius* could aggregate blood plates and significantly reduce blood glucose levels in mice [105,123]. Authors Ozioma et al. (2014) investigated anti-hyperglycemic properties and antioxidant enzymes with the ethanolic extract of *C. olitorius* leaves orally administrated to streptozotocin-induced diabetic rats and obtained promising results [123]. The result indicate a high safety index (no toxicity or fatality) after oral administration of 5000 mg/kg body weight of the ethanol extract, and the extract reduced blood glucose levels dose-dependently and reduced superoxide dismutase, catalase activities, and liver function enzymes assayed ALT, AST, and ALP when compared with nontreated groups. A similar trend was noted in the methanol extract of aerial parts, which significantly decreased blood glucose levels, and the highest dose of extract had better antihyperglycemic activity than with the drug glibenclamide loaded in mice [105]. The IC_50_ for α-amylase and α-glucosidase was found to be 27.95 µg/mL and 41.64 µg/mL, respectively, with the methanolic extract of *C. olitorius* exhibiting good antidiabetic potential [115].

A group of researchers investigated the antiobesity of *C. olitorius* leaf polyphenolic compounds in LDLR (low-density lipoprotein receptor), demonstrating that the effect was associated with a decrease in oxidative stress and an increase in β-oxidation in the liver [16]. The gene expression of the PPARα and CPT1A regulatory molecule in fatty acid β-oxidation significantly up-regulated by 1.56 and 1.61 fold, respectively, in the treated group, while gp91phox, a key subunit of NADPH oxidase and involved in oxidative stress, was significantly down-regulated (−1.61-fold) compared with the control group. The finding suggests that leaf extract possesses an anti-hyperglycemic effect, which is linked to managing diabetes mellitus and metabolic disorders in animals and helpful in preventing obesity caused by diet. In another study, researchers found that the *C. olitorius* leaf extract significantly reduced weight gain and visceral white adipose tissue in obese rats [124]. The levels of serum glucose, triglycerides, total cholesterol, lipoprotein-low cholesterol (LLC), free fatty acids, IL-1β, inflammatory cytokines (TNF-α and IL-6), insulin, HOMA-IR index, and leptin were significantly reduced in obese rat groups, along with a significant increase in high-density lipoprotein cholesterol (HDL-C) and the number of adipocytes. The findings coincide with other reports that the treatment of diabetes and obesity-accompanying insulin resistance and metabolic disorders is the most responsible component of phenolic compounds as well as phytol, including terpenes and phenolic compounds [125]. Therefore, the authors suggest leaf extract is effective as orlistat in preventing and ameliorating obesity, steatosis, fatty liver, hyperlipidaemia, and insulin resistance in rats fed a high-fat diet (HFD) by suppressing pancreatic lipase activity, TNF-α, IL-1β, and leptin resistance along with the up-regulation of adiponectin.

A group of researchers studied the effect of *C. olitorius* in structural changes of testicular tissue in streptozotocin-induced diabetic rats [126]. They found that blood glucose levels significantly decreased in the treatment groups compared with the diabetic group; seminiferous tubule degenerations were prevented, and apoptotic cell numbers were reduced. Results indicate that *C. olitorius* decreases the complications of diabetes mellitus-induced rat testis, and precise information of the experiment indicates possible therapeutic effects in diabetes treatment. A group of researchers demonstrated, significantly ameliorating inflammation-mediated obesity in high-fat diet mice that is associated with the inactivation of inflammatory reactions in gut serum, as well as 3T3-L1 cells, and an inhibition of metabolic endotoxemia and colonic inflammation as well as a rebalance of gut microflora, including an increase in Lactobacillus and decrease in Desulfovibrio [127]. It has been reported that alcoholic and chloroform extracts of *C. olitorius* (100 mg/kg) protected against diabetic complications such as hyperlipidemia and determinant effects on the liver tissue. These pharmacological effects are due to the presence of some bioactive phytochemical constituents such as theophylline, trans-2, 3-dimethoxycinnamic acid, 7-hydroxy-4-methyl coumarin, apigenin 7-glucoside, and glycitein [128].

### 5.7. Hepatobiliary, Renal, and Haematological Activity

This key metabolic organ detoxifies toxic metabolites, synthesizes proteins, and produces essential compounds required by the body for proper functioning [129]. In diabetes mellitus, impaired liver function is associated with decreased hepatic delta-aminolevulinic acid dehydratase (δ-ALAD) activity. In a recent study, it was reported that water-soluble extracts of *C. olitorius* leaves ameliorated the alcohol-induced increase in hepatic inflammation and lipogenic protein levels by improving the gut–liver axis [50]. Saliu et al. (2019) reported that supplementation of jute leaf (*C. olitorius*) to streptozotocin-induced diabetes rats at 100 mg/g for 30 days significantly (*p* < 0.05) restored the decreased hepatic δ-ALAD activity and increased hepatic catalase and SOD activity accompanying the decrease in serum AST and AST activities [130]. Thus, the finding suggested that *C. olitorius* leaf extract has hepatoprotective properties and could protect the liver from diabetes-induced oxidative damage as well as improve the activities of some critical enzyme systems required for normal hepatic function, such as delta-aminolevulinic acid dehydratase. Laskar et al. (1986) reported that after feeding a protein-enriched diet made from *C. olitorius* seeds bodyweight, including the weight of the liver, increased in test animals and also significantly increased AST, ALT, and total lipid of the liver, whereas the AST and ALT of serum decreased. It was found that a cholesterol-free diet containing powdered green leaves of *C. olitorius* in rats helps to reduced hepatic cholesterol and increased fecal bile acid via inhibition of enterohepatic circulation condition and neutral sterol excretion [131]. Earlier studies discovered that rats with hypercholesterolemia had lower levels of serum and hepatic cholesterol and increased fecal steroid excretion [132], where rats fed a diet JML (fridze dried leaf extract) containing a water-soluble viscous polysaccharides containing dietary fibers are the principal effective component.

A study conducted by Mazumder et al. (2003) observed the impacts of different doses of *C. olitorius* on liver, kidney, and hematological parameters [133]. Results point out high doses significantly increased WBC counts, whereas SGOT, SGPT, NPN, and plasma cholesterol levels increased to their highest levels when given in medium and high doses. A group of authors demonstrated that the aqueous extract of *C. olitorius*, after administration for 30 days on haematological and plasma biochemical parameters in male albino rats, was found to have no significant effect of PCV, RBC, MCV, MCHC, platelet, neutrephil, lymphocyte, oenophile, and monocyte values at various doses in their respective tests, but the TWBC count was substantially decreased compared with the controls [134]. So, it may be recommended for people with blood disorders to consume. A histopathological study supported that AECO markedly mitigated chemically induced liver toxicity and preserved the histo-architecture of hepatic tissue [135]. Do et al. (2021) reported that the water-soluble extract of *C. olitorius* leaves ameliorated the alcohol-induced increase in hepatic inflammation and lipogenic protein levels by significantly down-regulating the expression of inflammatory cytokines, improving the gut microbial composition that helps to improve gut health [50].

### 5.8. Anticonvulsant Activity

Methanolic extract of *C. olitorius* seed (MECO) provided significant protection against chemo-convulsive agent-induced convulsions in mice by altering catecholamine and brain amino acid levels [133]. Results suggest significantly increased catecholamine levels in extract-treated mice, and GABA (γ-amino butyric acid) increased in mice brains after six-week treatment. Along with GABA, increased glutamate resulting from conversion of α-ketoglutarate to the glutamic transmission of glutamine levels may be associated with MECO’s anticonvulsant properties. So, it has been suggested that extract processed significant roles in CNS depressant and anticonvulsive properties against chemoconvulsions in mice, probably due to increased catecholamines and 5-HT in mice. Das and his colleagues figured out how to handle *C. olitorius* leaves’ aqueous extract cardiotoxicity mediated against sodium arsenite in experimental rats [136], suggesting leaf extract has a significant defense effect against myocardial injury caused by arsenic.

### 5.9. Neuromodulatory Activity

Dietary polyphenols are gaining scientific attention for their protective effects against degenerative diseases and cognitive decline due to its antioxidants properties [137]. A group of researchers revealed that hydroalcohol extract of *C. olitorius* leaves had inhibitory effects on neuroinflammation in a mouse model of lipopolysaccharide (LPS)-induced neuroinflammation [138]. They characterized 25 metabolites, and one of the major constituents (5.7%) was 1,5-dicaffeoylquinic acid, which significantly protected microglia from H_2_O_2_-induced cytotoxicity. The LPS group showed stronger GFAP immunoreactivity when compared with the control group and reduced GFAP expression in Co extract in the pretreated group compared with the LPS group. Immunohistochemistry showed decreased expression of the astrocytic marker glial fibrillary acidic protein, which is linked to inflammatory activity, and the inflammatory marker cyclooxygenase-2, all of which support the findings of previous research. These activities indicate several polyphenols such as mono- and dicaffeoylquinic acid that protect MC65 cells from Aβ-induced cell death and lead to controlling oxidative stress, in addition to other effects such as anti-Aβ fibrillation, and delay the aging process as well as neurogenerative disorder development [139,140]. Findings of the study correlated with significant improvement of cognitive function as well as abatement of GFAP and COX-2 expression and reduction in LPS-induced neurodegeneration in Co-treated mice. This may be due to the abundance of flavonoids in *C. olitorius* leaves and demonstrated neuroprotective properties by increasing neuronal viability, decreasing dopaminergic neuron loss in the brain, acting as an antiamyloidogenic agent, and ameliorating cognitive impairment [141]. Therefore, phenolic compounds have achieved attention in the medicinal field due to lowering risk by altering the progression of multifactorial diseases such as neurodegenerative and cardiovascular diseases and cancer.

### 5.10. Antioesterogenic and Antifertility Activity

It has been found that the methanolic extract of *C. olitorius* seed arrested the normal estrus cycle in female mice, and tremendously delayed sexual maturation was proven by the age at the vaginal opening and first oestrus appearance [142]. On administration of the methanolic extract of *C. olitorius*, the decreased weight of ovaries and uterus was observed in the adult. In treated mice, the content of cholesterol and ascorbic acid in the ovaries significantly increased, and two key enzymes (∆^5^-3-β hydrxysteroid dehydrogenase (HSD) and glucose-6-phosphate dehydrogenase (G-6-PD) decreased significantly, along with the decreased weight of the ovary, uterus, and pituitary. On the basis of available data, it is hypothesized that depression and reduction in ovarian steroidogenesis are the causes of delayed maturation in mice treated by the extract of *C. olitorius* seed. The improved substrate and decreased enzyme concentration is an indication that steroidogenesis is inhibited, and it may be induced by the presence of flavonoids in *C. olitorius* leaf extracts [143].

On the other hand, the fertility test by MECO treated with medium and high dose in male mice showed 100% negative results, and different dose levels resulted in a simultaneous decrease in testicular Δ^5^-3β-hydroxyl steroid dehydrogenase and glucose-6-phosphate dehydrogenase activities, both of which are involved in testicular steroidogenesis [144]. In the testis of MECO-treated mice, the activities of ascorbic acid oxidase, lactate dehydrogenase, and malic dehydrogenase were decreased, whereas carbonic anhydrase activity was significantly increased. All of these findings suggest that the methanol extract of *C. olitorius* seed inhibited gonadal steroidogenesis and thus produced antifertility activity in sexually matured male mice. All of these findings suggest that MECO inhibited gonadal steroidogenesis, resulting in an antifertility effect in sexually mature male mice.

### 5.11. Dermatitis and Wound Healing Activities

Wound healing is the process by which the skin and other soft tissues are repaired following injury. Authors Yokoyama et al. (2014) investigated the skin hydration on atopic dermatitis in mice using *C. olitorius* leaf extract excluding high MW (COEW), and the results show a possible therapeutic benefit for atopic dermatitis due to decreased plasma immunoglobulin E (IgE) level as well as mast cell degranulation [145]. In this study, mice treated with the COEW cream experienced significantly increased skin hydration and reduced transepidermal water loss (TEWL) compared with control mice, but IgE concentrations were not affected, whereas NC/Nga mice showed a decreased AD score. It has been stated that *C. olitorius* leaves comprise high fatty acid counts, specially α-linolenic and linoleic acid, and high quantities of antioxidant molecules [31]; these compounds have the ability to reduce inflammation by suppressing activation of ROS and lead to the aggravation of AD [146,147]. According to Ohtani et al. (1995), polysaccharides are known to facilitate skin hydration, and *C. olitorius* leaves contain a significant amount of mucilaginous polysaccharides, providing adequate skin hydration and reducing skin dryness [20].

Similar studies have shown that the optical densities of the plasma of rats treated with different doses have decreased over time, suggesting *C. olitorius* has a platelet-aggregative influence [123]. A separate study stated that both plant powder and aqueous extracts of *C. olitorius* exhibited a high level of antioxidant activity and significant wound healing activity in an excision model [148], where on the 18^th^ day the percentage of wound contraction was determined to be 100% for powdered plant ointment, 5% for powdered plant ointment, and 100 mg/mL for aqueous extract, respectively. The microbial load on the wound surface was estimated similarly to the wound healing capability, and research findings indicated traditional uses of this plant for wound treatment. Induced wound healing and the regeneration of injured tissue characteristics, alongside avoiding side effects and resistance of synthetic medicines, mean *C. olitorius* could be an alternative source of wound healing medicine to develop.

### 5.12. Antimicrobial Activity

Phytochemicals are considered to be attractive sources of natural antimicrobial compounds for combating the future public health challenge of antibiotic resistance. In order to test the efficacy of *C. olitorius* leaf extracts as antimicrobials, two different assays were used: agar diffusion and tube dilution, and both aqueous and methanolic extracts towards several bacteria such as *Escherichia coli*, *Salmonella typhi*, *Klebsiella pneumoniae*, and *Staphylococcus aureus* have been well-reported, with methanolic extracts exhibiting greater inhibition and activity indices than aqueous extracts. Susceptibility increased with concentration, with *E. coli* exhibiting the greatest susceptibility. This can be explained by the existence of phytochemical elements and is prophylactically significant [149], and the choice of extraction solvents influences antimicrobial and antibacterial activities. Following that, the effect of three differently derived extractions (EE, AEE, and AE) of *C. olitorius* against five Gram-negative and three Gram-positive bacteria by Yakub et al. (2018) [53] suggests that both EE and AEE have the potential to fight all tested bacteria, as well as demonstrate a notable increase in the zone of inhibition with an increasing concentration of extract. EE had the highest zone of inhibition, followed by AEE and AE against all strains, where maximal inhibitions were noticed for *S. aureus* (34.5 mm), *K. pneumonia* (23.5 mm), and *M. luteus* (22 mm) at 50 mg/mL. It was also found that mucilaginous polysaccharides (PSc) extract had a broad spectrum of antibacterial activities against all (Gram^+^ and Gram^−^) bacteria tested, whereas soluble fraction (SF) extract was effective against *Salmonella enterica* and *Klebsiella pneumoniae* at a concentration of 25 mg/mL [41]. In another study, Rume et al. (2016) reported that the N-hexane fraction of a methanolic extract of *C. capsularis* leaves exhibited antibacterial and antifungal activity with a zone of inhibition of 0.9–1.5 mm, followed by the hexane extract.

The authors described the in vitro interaction of *C. olitorius* leaf extract with five antibiotics on methicillin-resistant and methicillin-sensitive Staphyloccus aureus [150]. The most remarkable activity was against all bacterial strains tested, specially *S. aureus and E. coli*, which were unable to grow in the presence of components of stem dry oil, while components of leaves and stem oil had antimicrobial properties with MIC values ranging from 0.40–0.8 and 1.6- > 3.2 mg/mL, respectively [70]. The study concluded that petroleum ether extracted leaves offered an excellent activity against *E. coli*, *S. aureus*, and *Y. enterocolitica*, 20 mm, 19 mm, and 19 mm, respectively.

Conditions of extraction and the type of solvent influence the jute-leaf antimicrobial activity, indicating the active compounds’ biochemical nature. Abir and other coauthors fractionated lipophilic leaf extracts of *C. olitorius* and *C. capsularis* were tested against *S. aureus* and *E. coli* along with a control organism, *E. coli* DH5± [151]. The results reveal that fractions of *C. olitorius* leaves were more effective against *S. aureus* (19 ± 2.80 mm inhibition zone), whereas fractions of *C. capsularis* leaves have been more effective against *E. coli* (15 ± 2.3 mm inhibition zone). Comparing the above findings, the antibacterial effects of *C. olitorius* leaf extract was shown to be higher than those of *C. capsularis* leaves. Additional analysis should be carried out on isolated fractions to introduce lead components of new-generation antibacterial drugs, which account for promising findings on antibacterial efficacy. In addition to antimicrobial activity, antiviral properties of C. olitorius extracts have been investigated. The aqueous layer of *C. olitorius* leaves against the measles virus was reported by a group of researchers [75].

### 5.13. Antimalarial Activity

According to Sathiamoorthy et al. (2007, 1999), an aqueous extract of *C. olitorius* exhibited strong growth of inhibition (>96%) against malaria parasite plasmodium falciparum [152]. In a recent study, the extract of *C. capsularis’* ability to control two common malarial vectors, *Anopheles stephensi* and a dengue vector *A. aegypti*, was reported [153], where *C. capsularis* ethyl acetate extract has been more prominent than acetone and methanol extracts in larvicidal activity. In terms of ovicidal activity, no hatchability was attained at 300–450 ppm in *An. Stephensi*, and 375–450 ppm in *Ae. Aegypti*, due to the action of phytochemicals present in the extract interfering with chorion of the eggs. A similar result was also recorded for the ethanolic extract of *Andrographis paniculata* against *An. stephensi* in ovicidal and gravid mortality [154]. Sathiyamoorthy also stated that aqueous leaf, stem, and seed extract of *C. olitorius* was found to have >96% growth inhibition against the malaria parasite *Plasmodium falciparum* [155]. The leaf extracts of *C. olitorius* were revealed to posses antimicrobial activity against different Gram-positive (*Enterrococcus fecealis* and *Bacillus subtilis*) and Gram-negative bacteria (*Escherichia coli*, *Proteus vulgaris*, *Klebsiella* spp., and *Serratia marcescens*) [156]. These finding suggest that *C. capsularis* and *C. olitorius* could be used to a greater extent to control and kill the mosquito menace. Since mosquitoes developed a resistance to all and altered the behavior of nontarget organisms, it is urgent to develop alternative sources of environmentally friendly drugs, preferably plants from nature.

### 5.14. Antinutrients and Insecticidal Effect

It has been reported that cyanide concentration in the fresh sample (128 mg/Kg) of *C. olitorius* was less than the maximum permissible level of 200 mg/kg of fresh weight vegetables [46]. This finding suggests that the cyanide concentrations in the fresh and pressed samples are insufficient to cause toxicity in humans. In other words, compared with processed samples, the total concentrations of nitrate, soluble oxalate, and oxalates in fresh leaves are higher than permissible levels. However, moderate cooking can effectively reduce plant toxins to acceptable levels without altering the plant’s nutritional potential.

In a separate study, Roy (2014) assessed the role of *C. capsularis* leaf phytochemicals in *Diacrisia casignetum* Kollar feeding, growth, and reproduction [157]. When compared with young and senescent leaf-fed insects, mature jute-leaf-fed insects appeared to have shorter larval and postlarval developmental durations but longer adult longevity (*p* < 0.05). The highest fecundity was observed on mature leaves, followed by young and senescent leaves. The result suggests that the growth and development of *D. casignetum* are associated with nutrient content in relation to the secondary metabolites of three types of jute leaves. It has been reported that consumption of higher quantities of secondary chemicals has been shown to significantly decrease adult longevity and fertility and delay larval growth [158]. It was concluded that mature leaves with high nutritional factors and low levels of total phenols and other secondary chemicals contributed to a shorter developmental period, increased growth rate, fecundity, and cumulative *D. casignetum*, followed by young and senescent leaves.

In a laboratory condition, the potency of *C. capsularis* petroleum ether extract of three stored product pests (*Callosobruchus chinensis*, *Sitophilus oryzae* L, and *Tribolium castaneum* Herbst) was evaluated in adult phase. It was pointed out that the highest efficacy was recorded against *C. chinensis* with LD_50_ (μg/cm) being 93.82 after 24 h and 5.63 after 48 h, followed by *S. oryzae*, while LD_50_ was 77.63 after 24 h and 23.32 after 48 h, with minimum performance against *T. castaneum* [159]. The results given support the findings of Mahfuz and Khanam (2007) [160], who stated that *C. capsularis* were extremely toxic to adults *T. confusum*.

### 5.15. Toxicology

*Corchorus* leaves have a long history of use in medicine and are widely assumed to be safe. However, the available toxicological data is limited, but it generally supports this assessment. In an As-contaminated rice-induced toxicities study, rats of groups II, III, and IV were fed standard laboratory pellets supplied with As-contaminated rice, 4% *C. olitorius* leaf powder and As-contaminated rice + 4% *C. olitorius* leaf powder, while Group I was nourished with laboratory pellets [21]. It was observed that Group II had significantly decreased Hb concentration, WBC and RBC count, and that platelet count decreased nonsignificantly compared with the control. Hematological abnormalities and other serum indices were significantly improved by *C. olitorius* leaves with arsenic-contaminated rice. The levels of TG, TC, and LDL-C were significantly higher in rats treated with As-contaminated rice (Group II) compared with controls. This change is most likely caused by increased proteolytic activity and/or decreased protein synthesis or destruction of hepatic protein-synthesizing subcellular structures. A possible explanation for the increased serum proteins could be the result of antioxidant activity of *C. olitorius* leaves, which comprise large amount of α-tocopherol equivalent Vit-E [8], which have an effect on hepatic insulin resistance and increase serum proteins by stimulating the incorporation of amino acids into protein [161]. A similar result was obtained by Adedosu et al. [162], where sodium arsenite elevated serum enzymes in rats, *C. olitorius* leaf extract restored them nearly to their control levels. A group of researchers also stated that aqueous *C. olitorius* leaf extract (AECO) significantly restores the biochemical and haematological parameters near to the normal status in Pb-acetate and CdCl_2_ intoxication-tested rats through antioxidant activity and/or by preventing bioaccumulation of Pb within the tissues of experimental rats [163,164]. Additionally to this, AECO could significantly reduce the Cd-induced increase in the Bad/Bcl-2 ratio and the over-expression of NF-jB, caspase 3, and caspase 9.

In another study, the authors demonstrated the effects of *C. olitorius* leaves (AECO) against sodium arsenite (NaAsO_2_)-induced cardiotoxicity in experimental rats [136]. The treatment of rats with AECO (50 and 100 mg/kg) for 15 days prior to NaAsO_2_ intoxication significantly protected cardiac tissue against arsenic-induced cardiac oxidative impairment, cardiac arsenic content, and DNA fragmentation. In addition, pretreatment significantly prevented the arsenic-induced alterations of lipid profiles as well as enhancement of MDA and protein carbonyl content, while GSH, GST, GPx, and GR significantly improved in cardiac tissue. Histological studies of the organs supported the protective role of jute leaves, probably due to the presence of the substantial quantity of antioxidants and its free radical scavenging properties of flavonoids and phenolic compounds. It is possible that supplementing with antioxidant-enriched *C. olitorius* leaves may be a potential therapy and directly connected to the amelioration of chronic Pb, As, and Cd intoxication. Thus, there is a tremendous opportunity to develop this vegetable as a dietary composition that contains nutritional value as well as free radical detoxification capability to help neutralize the negative effects of contaminants such as heavy metals, metalloids, and other pollutants.

Negm et al. (1980), concluded that seed extract was the most toxic preparation because it contains the highest percentage of cardiac steroids, followed by leaves, stems, and roots [79]. The leaf of *C. capsularis* contains HCN, which may be responsible for cytotoxicity. A lethal dose (LD_50_) of Corchoroside A in mice is 0.053–0.0768 mg/kg, and in the case of Corchoroside B, it is 0.059–0.1413 mg/kg [48].

**Table 4:** A summary of the biological activities of *C. capsularis* and *C. olitorius*.

Biological Activities	Species	Plant Parts	Extraction Solvent/Fed Diet	Main Findings	References
Antioxidant activity	*C. olitorius*	Leaf stem	96% alcohol	Both leaves and stem displayed DPPH radical scavenging (95.1% and 97.1%), respectively, at 400 µg/mL.	[70]
	,,	Leaf	Distilled water	Mucilaginous polysaccharides (PSc) showed more fantastic antioxidant activities than soluble fraction (SF) extract. Antioxidant activities were about 90% against DPPH•, 78% against lipid peroxidation, and 69% against β-carotene at 1.5 mg/mL.	[41]
Cardiovascular activity	*C. olitorius*	Seed	Methanol	Compounds such as Corchoroside A, Corchoroside B, Strophanthidin trioside, Coroloside, and Olitoriside are widely used for heart failure treatment.	[87]
Antitumor and anticancer promoting activity	*C. capsularis* *C. olitorius*	Leaf	Methanol	Phytol and mono galactosyldiacylglycerol at concentrations of 15 g/mL and 30 g/mL completely inhibited the induction of Epsterin-Barr virus (EBV) early antigen in Raji cells, and viable cells decreased about 20% in the inducer-treated Raji cells.	[17]
	*C. olitorius*	Leaf	Ethanol	ECO treatment had a dose-dependent effect on HepG2 cell proliferation and 12.5 µg/mL effectively triggered apoptosis by increasing caspase-9 activity mitochondria as well as caspase-mediated pathways.	[14]
	*C. olitorius*	Whole plant	Methanol	Cytotoxicity against HeLa, HL460 lung cancer cell line, and PC3 prostate cancer cell line, indicating antitumor potential and Galactolipid 1 antitumor promoting activity.	[13]
	*C. olitorius*	Leaf	Dichloromethane (DCM) and aqueous	Two extract-induced apoptotic in Colo-320 and Colo-741 cells lines at 50 µg/mL concentration, and extract-induced apoptosis was more effective in metastatic Colo-741, colon adenocarcinoma cell lines.	[12]
	*C. olitorius*	Stem	Dichloromethane	Extracts have growth-inhibiting effects on humans MCF-7 and MDA-MB-231 adenocarcinoma. Stigmasterol demonstrated cytostatic activity against Hep-2 and McCoy cells.	[45]
	*C. olitorius*	Leaf	67% methanol and chloroform	Polyphenol-enriched extracts (PEEs) of *C. olitorius* leaves significantly reduced the viability of tumor Caco-2 cancer cells without any detrimental effects on the healthy CCD841 line.	[49]
	*C. olitorius*	Leaf	Aqueous	Significant antiproliferative effects on SUIT-2, A-375 and AGS cells at a concentration as low as 2.54 mg/mL. Moreover, extracts strongly inhibited angiogenesis and the growth of A-375 and AGS tumors.	[94]
Antiulcer activity	*C.olitorius*	Leaf	Aqueous	Oral aqueous extract dose-dependently inhibited gastric ulcers. The extract (400 mg/kg) had the highest cure rate (94.08%) and (33.75%) for acetic acid and ethanol/aspirin-induced ulcers, respectively.	[97]
Antidiabetic and bioadsorbent properties	*C. olitorius*	Leaf	Methanol	The dose-dependent inhibition of α-amylase and α-glucosidase (12.5–50.0 µg/mL), as well as ACE (10.0–50.0 µg/mL), was observed.	[65]
	*C. olitorius*	Leaf		Phytol and terpenes have hepatoprotective and antiadipogenic properties and may help manage insulin resistance and metabolic disorders associated with diabetes and obesity.	[84]
	*C. olitorius*	Leaf	Methanol	The extract reduced blood glucose on a dose-dependent basis.	[105]
	*C. olitorius*	Leaf	Ethanol	Extract significantly reduced (*p* > 0.05) the activities of catalase, SOD, and liver function enzymes. Oral administration of extract had an anti-hyperglycemic effect in streptozotocin-induced diabetics, suggesting it could be used to treat diabetes mellitus.	[165]
	*C. olitorius*	Seed	Ethanol	Significantly reduced blood sugar levels in normoglycaemic, OGTT, and diabetic rats, as well as a suppressed postprandial increase in glucose-loaded rats, and a decreased blood glucose level in diabetic rats.	[32]
Antiobesity effect	*C. olitorius*	Leaf	Ethanol	It reduces oxidative stress and increases β-oxidation in the liver, which helps prevent diet-induced obesity.	[16]
		Stem Leaf	Dichloromethane	Oleanolic acid possesses glucose-lowering properties.	[45]
Antifertility activity	*C. olitorius*	Seed	Methanol	Seed extract inhibited male reproductive capacity in sexually mature mice, since it interferes with gonadal steroidogenesis.	[144]
Antinociceptive, anti-inflammatory, and antipyretic	*C. capsularis*	Leaf	Chloroform	Reduce the number of abdominal constrictions that was confirming its traditional use of inflammatory and pain-related diseases. It is also linked to curing chronic urinary bladder inflammation.	[29]
Anti-inflammatory and antipyretic	*C. olitorius*	Root	Distilled water	Extract significantly lowered the elevated temperature after the brewer’s injection. Compared with controls, approximately 50 mg/kg of active ingredient had the least granuloma weight.	[166]
	*C. olitorius*	Aerial part	Aqueous ethanol	Isoquercetin-rich extracts reduced carrageenan-induced rat paw edema in inflammatory exudates and demonstrated activity against metastatic melanoma, leukemia, and osteosarcoma cell lines.	[57]
	*C. olitorius*	Leaf	chloroform: methanol (2:1, *v*/*v*)	α-linolenic acid and linoleic acid ability to anti-inflammatory properties and wound healing promotion.	[72]
Proliferative activity	*C. olitorius*	Leaf	Chloroform and methanol	Polysaccharides rich in uronic acid showed proliferative activity toward the murine splenocyte.	[20]
Anticonvulsant activity	*C. olitorius*	Seed	Methanol	Seed extract significantly increased the level of catecholamines in mice brain after a 6-week treatment.	[165]
Gastroprotective activity	*C. olitorius*	Leaf	Ethanol	Extract administration significantly inhibited gastric wall mucus depletion, and a group treated with 400 mg/kg produced a significant amount of gastric mucus.	[100]
Hepatobiliary, renal and haematological activity	*C. olitorius*	Leaf	Leaf powder	Reduces hepatic cholesterol while increasing neutral fecal bile acid and neutral sterol excretion.	[131]
	*C. olitorius*	Seed	Powder	A significant increase was seen in AST, ALT, as well as total lipid of liver, while serum AST and ALT dropped.	[48]
	*C. olitorius Leaf*	Leaf	Water-soluble extract	Reduced the levels of serum biomarkers for liver injury and reduced the overexpression of inflammatory cytokines and lipid metabolism-related proteins in the liver.	[50]
	*C. olitorius*	Leaf	Leaf powder	Increased δ-ALAD, hepatic catalase, and SOD activities were observed in association with a decrease in serum AST and AST activity.	[130]
Wound healing	*C. olitorius*	Leaf	Methanol and aqueous	An excision wound model showed significant wound healing activity for both powder and aqueous extract.	[148]
Neuromodulatory activity	*C. olitorius*	Leaf	Ethanol	Extracts significantly correlated with improved cognitive function and reduced neurodegeneration induced by LPS.	[138]
Phytoalexin activity	*C. olitorius*	Fresh young leaf	95% ethanol	Isolated stress metabolites and volatile compounds had good activity against the microorganisms. Antifungal substances are released or fungioxic compounds are synthesized in response to infection or injury.	[84]
Antileishmanial activity	*C. capsularis*	Leaf	Chloroform	Showed potent antileishmanial activity against *L. donovani* promastigotes with an IC_50_ value of 79 μg/mL and exhibited very specific apoptotic features by targeting *Ld*TryR.	[80,167]
Antibacterial activity	*C. capsularis* *C. olitorius*	Leaf	Lipophilic extracts	Both of the species of *Corchorus* showed antibacterial activity against (Gram^+^ and Gram^−^) bacteria. Fractions of *C. capsularis* leaves extract were found to be more effective against *E. coli.*	[151]
	*C. olitorius*	Leaf	Petroleum ether, methanol, and ethyl acetate + water	Antibacterial or antifungal activity was observed in all extracts, and petroleum ether was demonstrated with zone diameters of 14 to 20 mm.	[166]
	*C. olitorius*	Leaf	Ethanol	Simultaneous administration of antibiotics to patients who eat *C. olitorius* regularly needs to be reappraised because of possible synergism and antagonism.	[150]
	*C. olitorius*	Seed	Petroleum ether, chloroform, methanol	Isolated cardenolide glycosides at 150 g/mL were the most effective against the bacteria tested, with a 20–25 mm zone of inhibition.	[168]
	*C. olitorius*	Leaf	95% ethanol	Volatile components are effective against Gram-positive and Gram-negative bacteria.	[84]
Antimalarial activity	*C. capsularis*	Leaf	Acetone, ethyl acetate, ethanol	Different extracts demonstrate mosquitocidal activity against *Anopheles stephensi*, a common malaria vector, and *Aedes aegypti*, a dengue vector.	[153]
Antimicrobial activity Antibacterial activity	*C. olitorius*	Leaf stem	96% alcohol	Leaf and stem oil components showed significant antibacterial activity with MIC values of 0.40–0.8 and 1.6- > 3.2 mg/mL, respectively.	[70]
	*C. capsularis*	Leaf	Methanol	The highest activities against Gram-positive, Gram-negative bacteria and fungi, with a 0.9 to 1.5 mm zone of inhibition.	[96]
	*C. olitorius*	Leaf	Methanol and aqueous	Both extracts had antimicrobial activities, but methanolic extracts displayed more comprehensive inhibition and activity indices.	[149]
Antifungal activity	*C. olitorius*	Leaf	Petroleum ether, methanol, and ethyl acetate + water	All extracts displayed varying antifungal activity, and ethyl acetate + water extract showed prominent activity against *Geotrichum candidum.*	[166]

## 6. Conclusions and Future Perspectives

In terms of nutritional value, the leaves of *C. olitorius* and *C. capsularis* are valued for their high dietary fiber, protein, vitamins, iron, and folate. Based on previous scientific reports on phytoconstitute and biological activities, *C. capsularis* and *C. olitorius* have potential to be used as a substitute ingredient in pharmaceutical and nutraceutical industries. Two species have various major phytoconstituents, namely chlorogenic acid, 3,5-dicaffeoylquinic, quercetin, flavone, tannins, diacylglycerol, uronic acid, α-linolenic acid, and β-sitosterol. Therefore, this justifies the therapeutic activity against a wide array of diseases due to the presence of numerous bioactive constituents and their antioxidant, anticancer, antiulcer, antidiabetic, antinociceptive, and antibacterial properties that can prevent a number of diseases. Biological mechanisms have been discovered for a small number of bioactive compounds, but many studies have shown that isolation and characterization of these compounds results in limited information. Additionally, the majority of these studies used crude extracts and, in some cases, fractions of the leaves without properly characterizing or standardizing the phytochemical constituents, which analyses of which might yield inconsistent or inaccurate results. Additional investigation into the specific phytochemicals present in *C. capsularis* and *C. olitorius* extracts may lead to the development of effective therapeutics for the prevention and/or treatment of these disease states. More research should be conducted to evaluate its usefulness as a means of preventing oxidative stress, cancer, ulcer, diabetes, inflammation, neuroinflammation, and microbial infections. Even with the promising findings obtained from the in vivo and in vitro studies, it is still important to conduct clinical trials that are randomized, controlled, and sufficiently large to obtain reliable therapeutic data. On the other hand, the mechanism of action of the chemical components and molecular mechanisms in combating different diseases or disorders should be explored by application of different techniques, such as genomics, transcriptomics, proteomics, and metabolomics. Based on the available literature and previously published scientific findings, it is proposed that both species have significant potential for human nutrition, health, and beauty care, as well as for development as functional ingredients due to their nutritional properties and bioactive compound composition.

## Figures and Tables

**Table 1:** Application of different parts as food and its ethnobotanical uses [8,18,28].

Species	Location	Part	Uses
*C. capsularis*	Bangladesh and India	Leaves along with petiole and tender shoots	Usually, sauteed lightly and eaten along with grains of rice or rice gruel.
	Malaysia	Leaves	known as “kancing baju”.
	Vietnam	Leaves	It is made into a soup with shrimp.
	Nigeria	Leaves	Used to prepare a stew called “ewedu”. Use jute leaves for treating iron and folic acid deficiency, as well as treatment of anemia.
*C. olitorius*	Northern Sudan, Tunisian, and Mali	Leaves	Used to make into a common medicinal mucilaginous (slimy) soup or stew.
	Egypt, Jordan, and Syria	Dried leaf, dried immature fruit	Ingredient as a soup-based dish, eaten as boiled vegetable with lemon and olive oil. Sometimes eat with meat over rice or lentis.
	Philippines	Leaves	Commonly eaten with bamboo shoots as a leafy vegetable.
	Japan	Leaves, dried leaves	Food item, thickener in soups, and young dried leaves as coffee/tea substitute.
	China	Seeds, dried leaves	Seeds are used as a flavoring agent, and dried leaves are used to made herbal tea.
	Europe		Leaves are used as soup.
	Middle East	Leaves	Green leafy vegetables and stews with rice.
	Kenya	Leaves	Leaves are eaten with “ugali”, a staple in most communities.
	Turkey and Cyprus	Young leaves	Generally cooked into a chicken stew.
	Thailand	Leaves	Eaten blanched, together with plain rice congee.
	Ghana, and Sierra Leone	Leaves	Complement of staple foods.

**Table 2:** Nutritional comparison of *C. capsularis* and *C. olitorius* mg per 100 g [8,40,46,47].

Ingredients and Unit	Leaves		Saluyot (Boiled/100 Grams Edible Portion)	Seeds of *C. olitorius*
	*C. capsularis*	*C. olitorius*		
Moisture (%)	80.4–84.1	86.30	-	0.006–0.011
Ash	2.4	8.11	4.5–5.6	1870–2642
Calories (kl cal)	73	37.6	43–58	-
Protein	3.6	26.80	4.5–5.6	970–1140
Crude fat	1.7–2.0	5.40	-	5270–5900
Carbohydrates 7.6–12.4	7.6–12.4	85	7600–12,400	59,910–87,340
Fiber	1.7–2.0	37.60	1700–2000	970–1910
Lipid	0.6	8460	-	-
Vit. A	-	-	6390	2.16–2.84
Thiamine	15	-	15	-
Riboflavin	28	-	28	-
Niacin	1.1–1.2	-	1.5	-
Ascorbic acid	75.80	257.8	95	0.52–0.88
Vit. D	-	-	-	0.36–0.39
*β*-carotene	6.41–7.85	5.44	-	-
α-tocopherol	-	14.0	-	-
Na	12	72.3	12	2.2–7.0
K	444	4.4	444	30–1230
Ca	298	-	266–366	1240–2610
Mg	33.66–37.99	34.35–37.84	5.95	910–1480
Fe	12.53	9.93–13.44	11.6	1240–1620
P	97–122	-	97122	0.62–0.97

**Table 3:** Chemicals isolated from different parts of *C. capsularis* and *C. olitorius*.

Chemical Classification	Compound Isolated	Plant Parts	Major Findings	Extraction Solvent	Method	References
Cardiac glycosides	*C. olitorius*	Seed and leaf	Strophanthidin glycosides and digitoxigenin glycosides	Methanol	HPLC	[85]
		Seed	Coroloside and deglycocoroloside			[48]
		Root	Corchoroside		Column chromatography	[58]
		Leaf	Capsulasone, cochorol, and capsularol			
		Seed	Caredenolide glycoside	Methanol		[86]
		Leaf	Ionone glucosides named corchoionosides A, B, and C, (65′,9Jt)-roseoside Two flavonol glucosides isoquercitrin and astragalin Two coumarin glucosides cichoriine and scopolin	Methanol		[36]
		Seed	Corchoroside A, corchoroside B, strophanthidin trioside, coroloside, deglycoroloside, oitoriside, glucoevatromonoside, deglucocoroloside, evatromonoside, and digitoxigenin triglycoside	Methanol		[87]
		Seed, root, leaf, and stem	Raffinose, coroloside, glucoevatromonoside erysimoside andolitoriside, and gluco-olitoriside	Chloroform	GC-MS	[4,65,86]
	*C. capsularis*	Seed	Strophanthidin glycoside, strophanthidin trioside, corchoroside A, corchoroside B, hydrogen cyanide, and polar glycosides A and B	Chloroform–alcohol (2:1)	Column chromatography	[58,88]
Cardenolide glycosides	*C. olitorius*	Seed	Canarigenin 3-*O*-B-D-glucopyranosyl-(1-4)-*O*-B-D allomethylpyranose/altromethylpyranose, cannogenol 3-*O*-β- D-glucopyranosyl-(1→4)-*O*-*β*-D-boivinopyranoside, periplogenin 3-*O*-*β*-D-glucopyranosyl-(1→4)-*O*-*β*-D- digitoxopyranoside, and digitoxigenin 3-β-D-glucopyranosyl-(1→6)-*O*-*β*-D-glucopyranosyl-(1→4)-*O*-*β*-D-digitoxopyranoside	Methanol	Medium pressure liquid chromatography and HPLC	[60,89]
Strophanthidin glycosides	*C. olitorius*	Seed	Erysimoside (strophanthidin 3-*O*-*β*-Dglucopyranosyl (1→4)-*O*-*β*-D-digitoxopyranoside), olitoriside (strophanthidin3-*O*-*β*-D-glucopyranosyl (1→4)-*O*-*β*-Dboivinopyranoside), corchoroside A (strophanthidin 3-*O*-*β*-D boivinopyranoside), and helveticoside (strophanthidin 3-*O*- β-D digitoxopyranoside),	Methanol	Medium pressure liquid chromatographyand HPLC	[61,89]
Digitoxigenin glycosides	*C. olitorius*	Seed	Glucoevatromonoside (digitoxigenin-3-*O*-*β*-D glucopyranosyl-(1→4)–*O*-*β*-D-digitoxopyranoside), coroloside (digitoxigenin-3-*O*-*β*-D-glucopyranosyl-(1→4)-*O*-*β*-D-boivinopyranoside), deglucocoroloside (digitoxigenin-3-*O*-*β*-D-boivinopyranoside), evatromonoside (digitoxigenin-3-*O*-*β*-D digitoxopyranoside), digitoxigenin 3-*O*-*β*-D glucopyranosyl-(1→6)-β-D-glucopyranosyl-(1→4)-*O*-*β*-D digitoxopyranoside, and corchorusoside (A, B, C, D, and E)	Methanol	Medium pressure liquid chromatography and HPLC	[61,89]
Triterpenes	*C. capsularis C. olitorius*	Root	Corosin (R_1_ = OH, R_2_ = R_3_= H), ursolic acid (R = H), corosolic acid, and oxo-corocin	Ethanol	Silicagel-colum chromatograph	[62]
	*C. olitorius*	Stem and leaf	Oleanolic acid	Dichloromethane	NMR spectra	[45,69]
	*C. olitorius*	Root	Corosic acid	Refluxing with hydrochloric acid		[65]
	*C. capsularis*	Leaf	Capsin (R_1_ = Glucose, R_2_ = H) and capsugenin 30-*O*-Glycopyranose (R_1_ = H, R_2_ = Glucose)	Methanol and sulphuric acid	TLC	[64,66]
	,,	Root	Betulin	Petroleum ether, chloroform, and methanol	column chromatography	[81]
Ursane tnterpenes	*C. capsularrs C. olltorius*	Root	Corosm (capsularone), ursohc acid, and corosohc acid	95% ethanol alcohol	TLC	[66]
Ionones	*C. olitorius*	Leaf	Corchoionoside-A, corchoionoside-B, corchoionoside-C (6*S*,9*R*)-roseoside, and Betulabuside	Methanol under reflux	TLC	[36,69]
Phenolics	*C. olitorius*	Leaf	Quinic acid, gallic acid, protocatechuic acid, 4-*O*-caffeoylquinic acid, caffeic acid, 1,3-di-*O* caffeoyquinic acid, *p*-coumaric acid, *trans*-Ferulic acid, 3,4-di-*O* caffeoyquinic acid, rosmarinic acid, and 4,5-di-*O*-caffeoyquinic acid	Ethanol, ethanol/aqueous, and aqueous	LC–MS	[53,57]
	,,	Leaf and seed	Quinic acid, chlorogenic acid, and 1,5-dicaffeoyl quinic acid 3,5-dicaffeoyl quinic acid	Petroleum ether Fraction	GC-MS	[36,90]
	,,	Dried leaf	chlorogenic acid, catechin, and astragalin	water-soluble extract	HPLC-ESI-MS	[54]
	,,	Leaf	5-caffeoylquinic acid (chlorogenic acid), 3,5-dicaffeoylquinic acid (isochlorogenic acid), quercetin 3-galactoside, quercetin 3-glucoside, quercetin 3-(6-malonylglucoside), and quercetin 3-(6-malonylgalactoside)	Methanol	NMR and FAB-MS	[47]
		,,	Chlorogenic acids, dicaffeoylquinic acids, and feruloyl-quinic acids	67% methanol and chloroform	UHPLCDAD-HRMS	[49]
		,,	Methyl-1,4,5-tri-*O*-caffeoyl quinate and trans-3-(4-Hydroxy-3 methoxyphenyl) acrylic anhydride	Methanol	NMR, IR, MS	[13]
		,,	Astragalin, isoquercetin, quercetin-3 galactoside, quercetin-3-(6 malonyl glucoside), and guercetin-3-(6 malonyl galactose)	Methanol	HPLC	[36,47]
		,,	Quinic acid	Ethanol	HPLC	[57]
	*C. capsularis*	Bark and leaf	Cyanidin and cyanidin glucoside	-		[48]
Flavonoids	*C. olitorius*	Leaf and seed	Quercetin, isoquercetin, astragalin, catechins, luteolin, and 3,5-dicaffeoylquinic acid (3,5-DCQA)	Petroleum ether fraction	GC-MS	[36]
	,,	Leaf	Naringin, apegenin-7-*O*-glucoside, quercetin (quercetin-3-*O* rhamonoside), kaempferol, naringenin, luteolin, apigenin, cirsiliol, and cirsilineol	Ethanol, ethanol/aqueous, aqueous	LC-MS	[53]
	,,	Leaf, aerial part	Astragalin (kaempferol 3-*O*-*β*-D glucopyranoside), tolifolin (kaempferol 3-*O*-β- D- galactopyranoside), and jugalanin (kaempferol 3-*O*-α-L-arabinopyranoside)	Methanol	NMR and FAB-MS	[47,65]
			Isoquercetin (quercetin 3-*O*-*β*-D-glucopyranoside)	Aqueous ethanol	HPLC	[57]
	*C. olitorius* *C. capsularis*	Leaves	Caffeic acid, *trans*-ferulic acid, rutin hydrate, ellagic acid, and quercetin hydrate	80% ethanol	HPLC-DAD	[54]
Flavonoid glycosides	*C. olitorius*	Leaf	Isoquercetin, astragaline, tolifolin and juglanin, oleanolic acid glyceryl monopalmitate, β-sitosterol, and β- sitosterol-3-glucoside	-		[65]
	,,	Aerial part	Quercetin3-*O*-galactoside (hyperoside), quercetin 3-β-glucoside (isoquercitrin), quercetin 3-(6 (malonylglucoside), and luteolin7-*O*-hexoside,	Hydroalcohol	HPLC	[90]
Coumarin glucosides	*C. olitorius*	Defatted seed	(4-,7-dihydroxy coumarin)	Chloroform		[59]
	*C. olitorius*	Leaf	Scopolin and cichoriin	Methanol	NMR	[36]
Sterols	*C. olitorius*	Leaf	β- Sitosterol-β-D-Sitosterol-glucoside, β-sitosterol 3-*O*-B-D-glucopyranoside	-		[65]
	,,	Stem and root	β- Sitosterol	Dichloromethane	NMR spectra	[45]
	,,	Stem	β-sitosteryl fatty acid esters, β-sitosterol, and stigmasterol	Dichloromethane	NMR spectra	[45]
	*C. capsularis*	Leaf	β-sitosterol	Chloroform	FTIR, HNMR, CNMR GC–MS	[80]
	,,	Seed, leaf, root and stem	β-Sitosterol	Dichloromethan, ethanol	Column chromatograph and Silicagel-colum chromatograph	[45,58,62]
	,,	Vegetative part	3-*O*-glucopyranosyl-β-sitosterol	Petroleum ether and alcoholic Potassium Hydroxide	GC-MS	[65]
Fatty acids	*C. olitorius*	Leaf Leaf	Corchorifatty acid-A, B, C, D, E, and F and undecanoic acid	Methanol	Silica-gel, HPLC	[69]
			Glyceryl monopalmitate			[65]
	,,	Leaf	α-linorenic acid and linoleic acid	Chloroform and methanol (2:1)	GLC	[72]
	,,	Leaf	ω3-octadecatriene ω-3 fatty acids/ω3-octadecatriene (49% of the total fatty acids)	Chloroform and methanol (2:1)	GCQTOF	[71]
	*C. olitorius*	Fresh young leaf	Scopoletin, fraxinol, isopimpinellin, xanthotoxol, and peucedanol	95% ethanol	GC-MS	[84]
	*C. olitorius*	Seed Stem	Stearic acid (49.48%)- seeds Palmitic acid (59.94%)- stems	Petroleum ether and chloroform	Gas–liquid chromatograph	[65]
	*C. capsularis*	Seed, vegetative part	Palmitic acid major in the seeds (81.68%) and the vegetative part (54.10%)			
Volatile component	*C. olitorius*	Leaf	In control and treated leaves, a total of 45 and 49 components were identified, with *cis*-3-hexen-1-ol, *cis*-4-hexen-1-ol, terpinolene, tetradecanal, sabinene, and phytol being the most abundant findings.	95% ethanol	GC-MS	[84]
Polysaccharides and other sugar	*C. olitorius*	Leaf	Uronic acid (65%) and composed of rhamnose, glucose, galacturonic acid, and gluconic acid.	Chloroform/Methanol	Chromatology	[20]
	*C. capsularis* *C. olitorius*	Seed	Free sugars, glucose, sucrose, fructose, raffinose, arabinose, and galactose	Methanol in ethyl acetate	Silicagel-colum chromatograph	[62]
	*C. capsularis* *C. olitorius*	Root	Glucose, fructose, arabinose, and raffinose	Methanol in ethyl acetate	Silicagel-colum chromatograph	[62]
	*C. capsularis*	Seed	Sucrose, raffinose, stachynose, and verbascose			
	*C. capsularis*	Leaf and bark	Fructose and galactose	-		[48]
Essential oil	*C. olitorius* *C. capsularis*	Leaf	Cedrane-5-one and γ-terpinene are major components of each species, respectively	Methylene chloride	GC-MS	[65]

## Abbreviations

ABTS	2,2’-azino-bis(3 ethylbenzothiazoline-6-sulphonic acid)
AFB1	Aflatoxin B
δ-ALAD	Hepatic delta-aminolevulinic acid dehydratase
CPT1A	Carnitine palmitoyl transferase 1A
COX-2	Cyclooxigenase-2
DAG	Diacylglycerol
DCM	dichloromethane;
DPPH	2,2-diphenyl-1-picrylhydrazyl
DW	Dry weight
EC_50_	Half maximal effective concentration
EGFR	Epidermal growth factor receptor
FFA	Free fatty acids
FRAP	Ferric reducing ability of plasma
GABA	γ-amino butyric acid
G-6-PD	Glucose-6-phosphate dehydrogenase
GPx	Glutathione peroxidase
GSH	Glutathione
GST	Glutathione S-transferase
HE	Hydrophilic extract
HFD	High-fat diet
HPLC	High-performance liquid chromatography
HDL-C	High-density lipoprotein cholesterol
HepG2	Human hepatocellular carcinoma
IC_50_	Half maximal inhibitory concentration
IgE	Immunoglobulin E
ICAD	Inhibitor of caspase-activated DNase
LDLR	Low-density lipoprotein receptor-deficient
LE	Lipophilic extract
LLC	Lipoprotein-low cholesterol
LPS	Lipopolysaccharide
iNOS	Inducible nitric oxide synthase
LC-MS	Liquid chromatography-mass spectrometry
LPS	Lipopolysaccharide
MAPK	Mitogen-activated protein kinase
MECO	Methanolic extract of *C. olitorius*
NMR	Nuclear Magnetic Resonance
PARP	Poly ADP-ribose polymerase
PEEs	Polyphenol-enriched extracts
PGE2	Prostaglandin E2
PSc	Polysaccharides
PTS	Phytosterol
PPARα	Peroxisome proliferator activated receptor alpha
QE/g	Quercetin equivalent per gram
ROS	Reactive oxygen species
SGOT	Serum glutamic oxaloacetic transaminase
SGPT	Serum glutamic pyruvic transaminase
SOD	Superoxide dismutase
STAT	Signal transducer and activator of transcription
TAG	Triacylglycerol
TFC	Total flavonoids content
T2DM	Type 2 diabetes mellitus
